# Comparative Transcriptome Analysis of Hard and Tender Fruit Spines of Cucumber to Identify Genes Involved in the Morphological Development of Fruit Spines

**DOI:** 10.3389/fpls.2022.797433

**Published:** 2022-03-15

**Authors:** Duo Lv, Gang Wang, Qi Zhang, Yao Yu, Pei-Chao Qin, Jin-An Pang, Jing-Xian Sun, Ke-Yan Zhang, Huan-Le He, Run Cai, Jun-Song Pan

**Affiliations:** ^1^School of Agriculture and Biology, Shanghai Jiao Tong University, Shanghai, China; ^2^Committee of Agriculture and Rural Areas of Jinshan District, Shanghai, China; ^3^Tianjin Derit Seeds Company Limited, Tianjin, China

**Keywords:** cucumber, trichomes, fruit spines, transcriptome analysis, morphological development, C-type lectin receptor

## Abstract

The spines of cucumber fruit not only have important commercial value but are also a classical tissue to study cell division and differentiation modes of multicellular trichomes. It has been reported that *CsTs* (C-type Lectin receptor-like kinase) can influence the development of fruit spines. In this study, we took a pair of cucumber materials defined as hard (Ts, wild type) and tender spines (*ts*, mutant) and defined the developmental process of fruit spines as consisting of four stages (stage I to stage IV) by continuously observing by microscope and SEM. Comparisons of transcriptome profiles at different development stages of wild-type spines showed that 803 and 722 genes were upregulated in the stalk (stage II and stage III) and base (stage IV) development stages of fruit spines, respectively. The function analysis of DEGs showed that genes related to auxin polar transport and HD-ZIP transcription factor are significantly upregulated during the development of the stalk. bHLH transcription factors and cytoskeleton-related genes were significantly upregulated during the development of the base. In addition, stage III is the key point for the difference between wild-type and mutant spines. We detected 628 DEGs between wild type and mutant at stage III. These DEGs are mainly involved in the calcium signaling of the cytoskeleton and auxin polar transport. Coincidentally, we found that CsVTI11, a factor involved in auxin signal transmission, can interact with CsTs *in vivo*, but this interaction does not occur between CsVTI11 and Csts, further suggesting that *CsTs* may regulate the development of fruit spines by influencing cell polarity. These results provide useful tools to study the molecular networks associated with cucumber fruit spine development and elucidate the biological pathways that C-type Lectin receptor-like kinase plays in regulating the development of fruit spines.

## Introduction

Trichomes are hairy structures covering the epidermis of plants. As a type of tissue that is in contact with the environment, they play an important role in resistance to biological and abiotic stresses (Johnson, 1975; Bennett and Wallsgrove, 1994; Mauricio and Rausher, 1997; Wagner et al., 2004). Based on their morphological characteristics, trichomes can be divided into several forms: unicellular or multicellular, glandular or glandless, and branched or unbranched (Werker, 2000). Therefore, trichomes provide an excellent model for studying cell differentiation and proliferation (Szymanski et al., 2000; Hülskamp, 2004).

*Arabidopsis* is a classical model plant, and studies on its trichomes are extensive in scope and number. Trichomes of *Arabidopsis* are single-cell structures; they originate from epidermal cells and go through six stages of development (Szymanski et al., 1998; Johnson et al., 2002) eventually distributing widely on leaves, stems, and sepals (Pesch and Hülskamp, 2004). The cell differentiation of trichomes in *Arabidopsis* is regulated by a series of competing transcription factors (TFs). R2R3-MYB transcription factor GLABRA1 (GL1; Oppenheimer et al., 1991), two basic helix–loop–helix (bHLH) proteins GLABRA3 (GL3) and ENHANCER OF GLABRA3 (EGL3; Payne et al., 2000), and a WD-repeat protein TRANSPARENT TESTA GLABRA1 (TTG1; Galway et al., 1994) can form a MYB–bHLH–WD40 trimeric complex, which can regulate the initiation of trichome by activating the downstream gene GLABRA2 (Rerie et al., 1994; Masucci and Schiefelbein, 1996; Szymanski et al., 1998). Some negative factors are also involved in the trichome developmental process. The single-repeat MYB proteins TRIPTYCHON (TRY; Martin and Martina, 2011), CAPRICE (CPC; Schellmann et al., 2002), ENHANCER OF TRY AND CPC1 (ETC1), ETC2, and CAPRICE-LIKE MYB3 (Kirik et al., 2004a; Simon et al., 2007) act partially redundantly as negative regulators, and they can inhibit the activity of MYB–bHLH–WD40 by competing with the R2R3 MYB protein to bind the GL3 (Kirik et al., 2004b; Wester et al., 2009).

Cucumber (*Cucumis sativus* L) is an annual species that is commercially important worldwide. According to a previous study (Zhao et al., 2015b), unlike *Arabidopsis*, cucumber has glandular and non-glandular multicellular trichomes at the same time. The non-glandular multicellular trichomes are visible throughout the aboveground part of the cucumber plant, especially on the fruit, where they are also known as fruit spines (Zhao et al., 2015a; Zhang et al., 2019; Du et al., 2020). Fruit spines are also an important agronomic trait that affects commercial value. Compared with that of the trichomes of other tissues, the structural differentiation of the fruit spines is more obvious. The basic structure of cucumber trichomes and fruit spines can be divided into a plinth of many cells and a stalk of single cells joined together. To date, some key genes involved in the development of cucumber trichome fruit spines have been identified. CsTril/CsGL3 is a class IV homeodomain-leucine zipper (HD-Zip IV) transcription factor that can activate the initiation of trichomes and fruit spines. So far, multiple allelic mutations of *CsTril*/*CsGL3* have been found. *Tril* (trichome-less) is a glabrous mutant that has a long segment insertion following the first exon of the *CsTril* (Wang et al., 2016). *gl3* (*glabrous 3*) is also a glabrous mutant, which has two types of mutation. The first type has three single-nucleotide polymorphisms (SNPs) in the fourth exon of the *CsTril* (Cui et al., 2016), and the second type has a retrotransposon insertion in the fourth exon (Pan et al., 2015). CsMict/CsGL1/TBH (Micro-trichome) is a member of the class I homeodomain-leucine zipper (HD-Zip I) family, and it can affect the density and development of cucumber trichomes and fruit spines (Li et al., 2015; Zhao et al., 2015b; Zhang et al., 2021). The results of genetic analyses and transcriptome profiling indicated that *CsTril* had an epistatic effect on *CsMict* during trichome development (Zhao et al., 2015c; Wang et al., 2016). CsMict can also interact with CsTTG1, a homologous protein of TTG1, to affect the number and size of the spines in cucumber (Chen et al., 2016). One C2H2 zinc finger transcription factor CsTu (tuberculate fruit) can affect the development of tubercules under the fruit spines by regulating CTK (Cytokinin) biosynthesis (Yang et al., 2014). CsTs (*tender spines*), a C-type lectin receptor-like kinase (LecRLK), can affect the structure of trichomes and fruit spines. A SNP (single-nucleotide polymorphism) in *Csts* leads to the loss of the first exon in the transcript and further causes cells from the basal part of trichomes to be arranged out of order from the normal style of the wild type (Wt; Guo et al., 2018), which indicates that *CsTs* is a key gene determining the differentiation of cucumber trichomes and fruit spines cells. To the best of our knowledge, there has been no detailed characterization of the developmental regulation of cucumber spines. In this study, we explored the developmental process of cucumber fruit spines in a wild type and a mutant (Mu) of CsTs, tender spines (*Csts*), and compared these processes. We further performed comparative transcriptome profiling analyses to identify genes and gene networks that might be involved in cucumber fruit spine development.

## Materials and Methods

### Plant Materials

The North China type “NC072” (wild type) and its soft fruit spine mutant “NC073” were provided by the Tianjin Derit Seeds Company Ltd. (Tianjin, China); NC072 and NC073 share almost the same genetic background. Cucumber plants were grown with appropriate management in a greenhouse (Shanghai, China) in the autumn of 2019 under natural photoperiodic conditions. Fruit spines from wild-type and mutant cucumber fruits that were 0.2, 0.35, and 0.6 cm in length were isolated under a dissecting microscope, and spines from at least 15 fruits from different plants were pooled as one biological sample. Three biological replicates were used for each set of experiments.

### Morphological Observation of Fruits and Spines

Cucumber fruits with a length of 0.2, 0.35, and 0.6 cm, as well as roots and receptacles, were fixed in FAA and vacuumed for 20 min then dehydrated through a gradient ethanol series (50, 60, 70, 85, 90, 95, and 100 % (v/v)) for every 5 min. They were then subjected to critical-point drying using the Leica EM CPD300 desiccator (Feica, Germany) and gold–palladium coating using the Leica EM SCD050 ion sputter and carbon coating unit (Feica, Germany). Finally, all samples were observed under an ALTO 1000 scanning electron microscope (Gatan, United States). Digital photos of cucumber fruits of different lengths were taken using a Canon EOS digital camera. Microscopy photos were taken using a LECIA DM 2500 microscope.

### Total RNA Extraction, Library Construction, and Illumina Sequencing

Total RNA was extracted using an RNeasy Plant Mini Kit (CWBIO, Beijing, China). RNA quantity and quality were analyzed using an Agilent Bioanalyzer 2100 system (US). The high-quality RNA samples were sequenced by Biomarker Bioinformatics Technology Co., Ltd. (Beijing, China), and the cDNA library used high-throughput sequencing (RNA-seq) with the Illumina HiSeq™ 2500. These raw data were deposited in the Sequence Read Archive (SRA) on the NCBI website. The accession number is PRJNA660492.^1^

### Sequence Assembly for RNA-Seq

The raw reads were first filtered to identify clean reads by removing the reads with only adaptor sequences and >5% unknown nucleotides, as well as low-quality reads (percentage of low-quality bases with a quality value ≤5 in more than 50% of a read). Clean reads were mapped to the cucumber genome sequence (cucumber 9930 genome v2; http://ftp.ensemblgenomes.org/pub/plants/release-51/fasta/cucumis_sativus/dna/ using HISAT2 software).^2^ Then, based on the reference genome sequence, StringTie software (Pertea et al., 2015) was used to join the mapped reads. If a read maps to an unannotated transcription region and encodes a peptide chain larger than 50 amino acid residues, it will be defined as a new gene.

### Differential Expression Genes Analysis

Gene expression levels were analyzed using fragments per kilobase of the transcript per million mapped reads (FPKM) method (Florea et al., 2013). Differential expression analysis of two samples was performed using edgeR (Robinson et al., 2010). edgeR provides statistical routines for determining differential expression in digital gene expression data using a model based on the negative binomial distribution. The resulting *p*-values were adjusted using the Benjamini and Hochberg approach for controlling the false discovery rate (FDR). Genes with an adjusted value of *p* <0.05 and fold change ≥1.5 found by edgeR were chosen as the thresholds for defining DEGs.

### Functional Analysis of DEGs

The functions of DEGs were identified using BLASTX (Altschul et al., 1997) against several classic biological databases, including the NCBI nonredundant protein database (NR http://www.ncbi.nlm.nih.gov), Gene Ontology database (GO, http://www.geneontology.org/), Kyoto Encyclopedia of Genes and Genomes database (KEGG, http://www.genome.jp/kegg/), Swiss-Prot database,^3^ Clusters of Orthologous Groups database (COG, http://www.ncbi.nlm.nih.gov), and Pfam database,^4^ using a cut-off E-value of 10^−5^. Then, we assigned DEGs to KEGG pathways using KOBAS 2.0 software (Mao et al., 2005). TopGO software^5^ was used to plot GO functional classification for the DEGs with GO term hits to view the distribution of gene functions of the species at the macro level. The analysis of homologous genes in our study used Blast function in NCBI and cucumber genome websites.^6^ When searching for homologous genes, we extracted the homologous genes with the same function and the highest homology.

### Yeast Two-Hybrid Assay by DUAL membrane System

We cloned the cDNA sequences of *CsTs* (without first 75 bp) and fused it into the pBT3-SUC vector. The ORF of *CsVTI11* was cloned and fused into the pPR3-N vector. All recombinant constructs were separately transformed into the yeast strain NMY51. Transformants were grown on SD media—Leu/-Trp and SD media—His/-Leu/-Trp/-Ade (with different concentrations of 3-AT). At least three independent experiments were performed, and the result of one representative experiment is shown. The operating details and functional testing of the DUAL membrane system refer to the studies of Toshiyuki et al. (2016) and Wu et al. (2019). The primers are listed in Supplementary Table S6.

### Bimolecular Fluorescence Complementation Assay

The full-length cDNA sequences of *CsTs*, *Csts*, and *CsVTI11* were cloned and fused with the pXY104 and pXY106 vectors (Yu et al., 2008; Liu and Howell, 2010), respectively. Tobacco (*N. tabacum*) leaves were used for co-expression studies as previously described (Schütze et al., 2009). The fluorescence signal was detected 2 days after infiltration using an Olympus BX 51 fluorescence microscope to acquire fluorescent images. YFP (yellow fluorescent protein) imaging was performed at an excitation wavelength of 488 nm. At least three independent replicates were performed, and the result of one representative experiment is shown. The primers are listed in Supplementary Table S6.

### Real-Time qPCR Analysis

To estimate the accuracy of RNA-Seq, we performed quantitative RT-PCR (qRT-PCR) using the same RNA samples that were used for RNA library construction. First-strand cDNA was prepared according to the PrimeScript RT Reagent Kit with gDNA Eraser (CWBIO, Beijing, China) protocol. qRT-PCR was conducted using FastStart Essential DNA Green Master Mix (Roche, Mannheim, Germany). *CsActin3* (*Csa6G484600.1*) was used as an internal control. qRT-PCR was performed in a total volume of 20 μl, containing 2 μl of cDNA, 10 μl of SYBR mix, 2 μl of gene-specific primers (10 μM), and 6 μl of ddH_2_O, using the CFX Connection Real-Time System (Bio-Rad, CA, United States) with 40 cycles of 5 s at 95°C and 30 s at 60°C. Three biological replicates were analyzed for each treatment, each biological replicate contained three technical replicates to qRT-PCR. Geneious software (version 2019.0.3) was used to design primers according to the cDNA sequences (Supplementary Table S6). The data from real-time PCR amplification were first analyzed using the 2^−△△CT^ method (Kenneth and Thomas, 2002).

## Results

### Developmental Stages of Cucumber Fruit Trichomes

**Figure 1:**
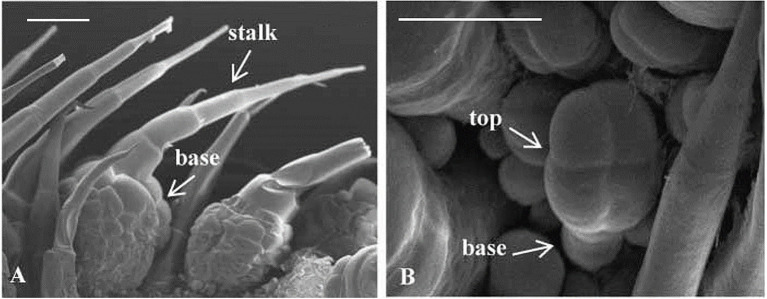
Morphological characterization of two types of trichomes of cucumber. **(A)** Type I trichomes, 200 μm. **(B)** Type II trichomes, 200 μm.

**Figure 2:**
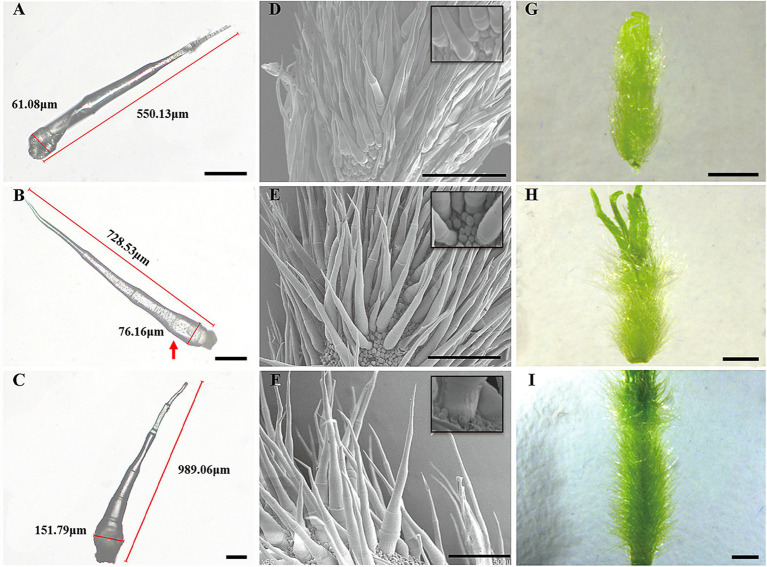
Key developmental stages of wild-type spines. **(A–C)** Light microscopy image, 100 μm; **(D–F)** SEM images, 500 μm; **(G–I)** Digital camera images under a microscope, 1 mm; **(A,D,G)** Stage II of spine development; **(B,E,H)** Stage III of spine development; and **(C,F,I)** Stage IV of spine development. The red arrow represents the fifth cell of the spines.

**Figure 3:**
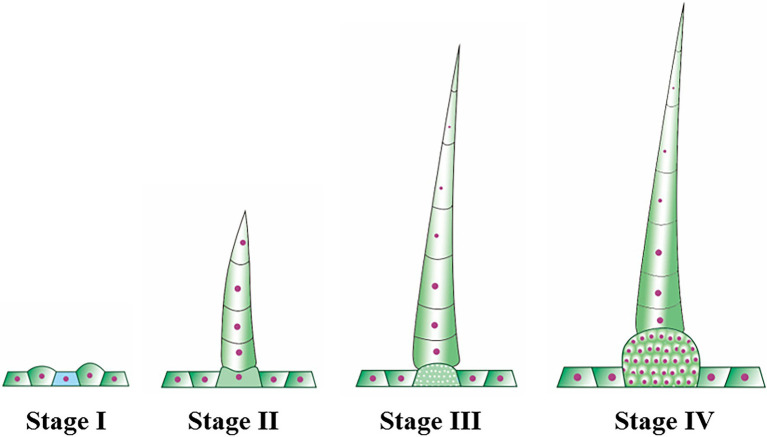
The schematic diagram of cucumber fruit spines at different developmental stages.

Trichomes were widely distributed on the leaves, tendrils, stems, calyx, and fruit of cucumber. The trichomes could be divided into two types. Type I was generally visible to the naked eye, and it had an obvious spiny structure (Figure 1A). The characteristics of type I trichomes included a pyramidal apical cell connected to a cylindrical structure consisting of 5–8 single cells, which formed the stalk. Below the stalk was a base made of hundreds of spherical cells. The structures of type I trichomes on different tissues were generally similar but type I trichomes on the cucumber fruit were larger and harder than those on other tissues, so they were called fruit spines. Contrary to type I trichomes, type II trichomes were much smaller, and their top was composed of four cells that were not very distinct from each other. This top was connected to the epidermis by 3–5 cells (Figure 1B). Since the fruit spines were more consistent than other type I trichomes on other tissues in the development state and their cells were harder and larger, making them convenient for slicing, we decided to focus on fruit spines as the research object in order to explore the developmental process of type I trichomes. Combined with the findings of previous studies (Zhao et al., 2015b), microscopy and SEM observations were used to divide the developmental process of spines into four stages (Figures 2A–I, 3).

In stage I, there were pus-like protrusions on the cucumber epidermis where the spines would eventually develop, and then, the apical cells developed first in these protrusions; in stage II (Figures 2A,D,G), the development of the stalk was initiated. The mode of development was as follows: the single cells that made up the stalk developed in the form of a relay; when one cell began to develop, the next cell was flat, similar to a cake. This “cake-like” cell would not begin to lengthen until the last cell had completely developed. This stage continued until the fruit grew to approximately 0.25–0.30 cm in length and the stalk of the spines had four complete cells. During stage I and stage II, the fruit was relatively thin, and because of the low rigidity of the newly developed spines, they clung to the fruit like “fetal hair,” while the population of cells that later developed into the base also underwent modest elongation but did not complete the initiation of development. At the start of stage III (Figures 2B,E,H), the fruit swelled obviously and grew to 0.35 cm in length. The fifth single-cell completed development, and the rigidity of the spines began to increase, causing the stalk to become erect. This stage continued until the fruit length was approximately 0.65 cm. The end of stage III was also the end of stalk development. The mature stalk was composed of 7–8 fully elongated single cells. Morphologically, the stalk was narrow at the top and wide at the bottom. The cells that developed later were larger than the ones that grew earlier, but the cell connected to the base (the last single cell) was shorter than other single cells, ensuring structural stability. In stage IV (Figures 2C,F,I), the population of cells that made up the base began to multiply and expand in volume until development was completed, forming a spherical pedestal.

### CsTs Influences the Development of Spines After Stage II

To determine the specific stages at which differences in spine form between the wild type (*CsTs*) and mutant (*Csts*) occurred, we continuously compared spine development state at different stages.

**Figure 4:**
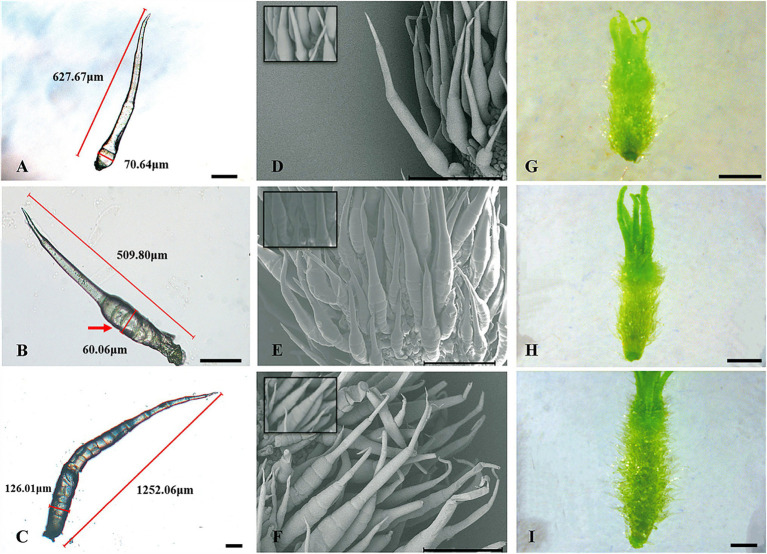
Key developmental stages of mutant (*Csts*) spines. **(A–C)** Light microscopy image, 100 μm; **(D–F)** SEM images, 500 μm; **(G–I)** Digital camera images under a microscope, 1 mm; **(A,D,G)** Stage II of spine development; **(B,E,H)** Stage III of spine development; and **(C,F,I)** Stage IV of spine development. The red arrow represents the fifth cell of the spines.

**Figure 5:**
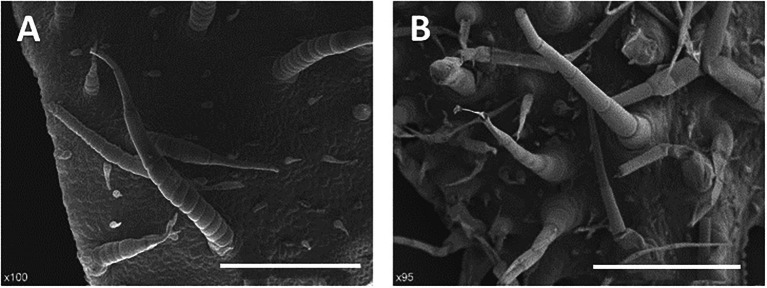
Morphological characterization of trichomes of receptacles. **(A)** Wild type, 500 μm; **(B)** Mutant (*Csts*), 500 μm.

**Figure 6:**
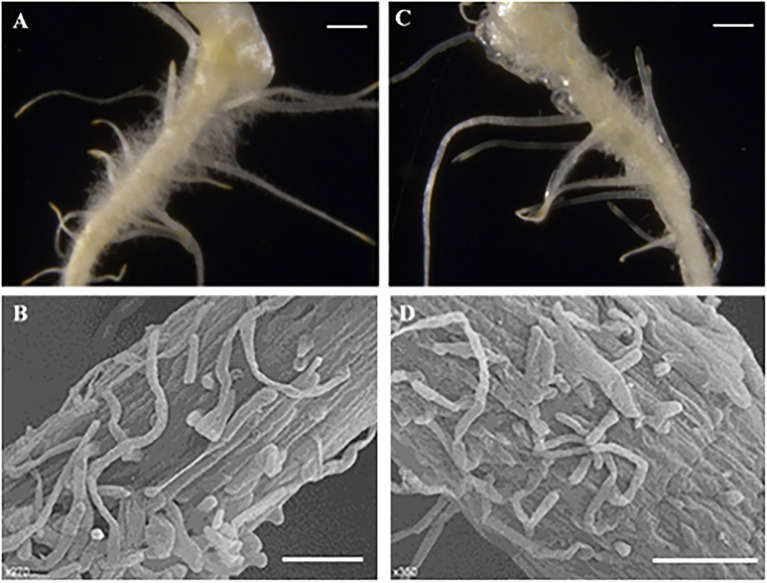
Morphological characterization of root hair. **(A–C)** Light microscopy image, 1 cm; **(B–D)** SEM images, 100μmn; **(A,B)** Wild type; and **(C,D)** Mutant (*Csts*).

In stages I and II (Figures 4A,D,G), the spine development state of the mutant was consistent with that of the wild type, and both had four complete single cells that clung to the fruit, similar to “fetal hair.” In stage III (Figures 4B,E,H), starting with the fifth cell, all single cells that made up the stalk were not fully elongated like those in the wild type; instead, they were stacked together like a multilayer pie. At the same time, the growth and division mode of single cells (after the fifth single cell) also changed, with some cells becoming shorter and some dividing into multiple cells. In the mutant, stage IV (Figures 4C,F,I) no longer seemed to exist, and the boundary between the base and stalk was no longer obvious. The base of the spines was no longer a spherical structure but an oval structure that linked a shorter stalk with the epidermis of the cucumber. The narrow lower part of the oval structure led to the unstable binding of spines on the epidermis, resulting in the spines of the mutant appearing to be soft and elastic. Finally, the mature spines were distorted due to disorder of the cell division mode, the spines were curved, and a large vacuole formed in the fifth single cell (Guo et al., 2018). From stage III, although the stalk (or spines) was erected due to an increase in its rigidity, the insufficient development of cells led to a disorganized arrangement of spines on the mutant fruit surface compared to the wild-type fruit surface. We also observed hairs on receptacles of the wild type and mutant and found that they also had this structural difference (Figure 5). However, no difference in root hair structure was found between the wild type and the mutant by either microscopy or SEM (Figure 6).

### Transcriptome Analyses of Cucumber Fruit Spines

To explore the gene networks involved in different stages of spine development in the wild type and mutant, we collected spines from three different developmental stages (stage II, stage III, and stage IV) for RNA-seq. RNA-seq was performed for three biological replicates in each stage. On average, approximately 5.94 Gb bases were generated from each sample by the Illumina HiSeq platform. After mapping sequenced reads to the reference genome (cucumber 9930 v2 genome), a total of 23,943 genes were obtained from all samples, including 695 new genes. The data quality of RNA-Seq is summarized in Table 1. We also calculated correlations between the samples from different stages and materials to test whether the samples chosen were reliable. The three replicates at the three stages showed a good correlation (Supplementary Figure S1).

**Table 1:** The summary of RNA-Seq data.

Samples		Clean reads	Clean bases (Gb)	GC content (%)	≥Q30 (%)	Total reads	Mapped reads (%)	Uniq mapped reads (%)	Multiple map reads (%)
Wild type (stage II)	a	19,868,313	5.94	45.13	92.25	39,736,626	95.18	91.49	3.69
	b	26,331,262	7.86	44.99	92.87	52,662,524	95.86	93.01	2.85
	c	23,626,477	7.06	44.93	93.11	47,252,954	95.93	93.06	2.87
Wild type (stage III)	a	24,889,139	7.44	45.01	92.79	49,778,278	96.15	93.56	2.59
	b	20,128,216	6.01	44.91	92.44	40,256,432	95.77	93.16	2.61
	c	20,749,728	6.20	44.89	92.87	41,499,456	95.78	93.17	2.61
Wild type (stage IV)	a	26,136,943	7.80	45.00	93.08	52,273,886	96.33	93.92	2.4
	b	23,890,024	7.12	45.01	92.95	47,780,048	96.22	93.98	2.24
	c	22,739,708	6.80	44.99	92.63	45,479,416	95.75	93.42	2.33
Mutant (stage II)	a	22,120,401	6.60	45.31	93.61	44,240,802	95.48	91.63	3.85
	b	19,907,633	5.95	44.95	92.75	39,815,266	95.67	93.13	2.53
	c	35,438,516	10.57	44.96	92.83	70,877,032	94.26	91.39	2.86
Mutant (stage III)	a	22,180,674	6.62	45.03	92.51	44,361,348	95.74	93.22	2.52
	b	24,050,730	7.18	45.00	92.6	48,101,460	95.72	93.11	2.61
	c	21,275,012	6.35	45.03	92.26	42,550,024	95.45	93.22	2.23
Mutant(stage IV)	a	24,924,007	7.44	44.97	92.4	49,848,014	95.64	93.43	2.21
	b	25,212,564	7.53	45.05	92.88	50,425,128	95.82	93.55	2.27
	c	25,757,883	7.69	44.97	92.24	51,515,766	94.53	92.15	2.38
Average		23,845,957	7.12	45.01	92.73	47,691,914	95.63	92.98	2.65

**Figure 7:**
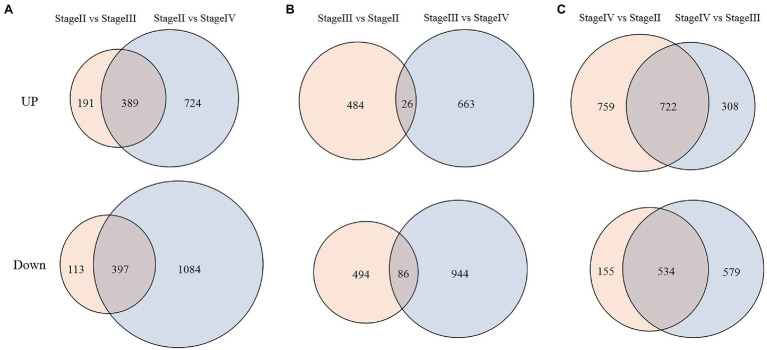
DEGs between different development stages of fruit spines in wild type. **(A)** DEGs at stage II compared with stage III and stage IV; **(B)** DEGs at stage III compared with stage II and stage IV; and **(C)** DEGs at stage IV compared with stage II and stage III. Up represents upregulated DEGs. Down represents downregulated DEGs.

Using the thresholds of a false discovery rate <0.05 and fold change of expression level ≥1.5, we divided the transcriptome data into two groups for analysis according to different explored directions by comparing fragments per kilobase of transcript per million fragments mapped (FPKM) values from different libraries (Supplementary Table S1). For group 1, we identified differentially expressed genes (DEGs) with expression specificity at different developmental stages of fruit spines in the wild type. As shown in Figure 7, expression levels of 389 genes were significantly upregulated at stage II compared with stage III and stage IV, while 397 genes were significantly downregulated at stage II compared with stage III and stage IV (Figure 7A). There were also 26 DEGs with the highest expression levels at stage III and 86 DEGs with the minimum expression level at stage III (Figure 7B). There were also 722 DEGs with the highest expression levels at stage IV and 534 DEGs that were significantly downregulated at stage IV compared with stage II and stage III (Figure 7C). For group 2, because stage III was the critical period for the difference between the wild type and mutant, we focused on DEGs that were not differentially expressed at stage II but were significantly differentially expressed at stage III between the wild type and mutant. A total of 628 genes showed this expression pattern in the transcriptome data, among which 249 genes were upregulated and 379 genes were downregulated in the mutant (Supplementary Table S1). Although the mutation of *CsTs* (*Csa1G056960*) is the main reason for the phenotypic difference between wild type and mutant, there was no significant difference in the expression level of *CsTs* between wild type and mutant. At the same time, the expression of *CsTs* in both the mutant and the wild type did not change during the three developmental stages of fruit spines (Supplementary Figure S2).

To validate the accuracy of the RNA-seq data, quantitative RT-PCR was conducted on 10 randomly selected DEGs from our RNA-seq data to detect their expression patterns at three development stages. These DEGs included *Csa3G748220* (*CsMict* from downregulated DEGs of mutant), *Csa5G577350* (*CsTu* from DEG-IVs), *Csa3G824850* (*CsMYB6* in DEG-IIs), *Csa2G006270* (ERF TF from DEG-II–IIIs), *Csa3G020060* (Aspartic proteinase from DEG-II–IIIs), *Csa1G006300* (signal response regulator from DEG-IIs), *Csa1G267240* (Chitinase from DEG-IIs), *Csa3168940* (MYB TF from DEG-IIs), *Csa3G824850* (MYB TF from DEG-II–IIIs), and *Csa5G623360* (Aquaporin from DEG-IIs). The results showed there was high expression pattern correlation between the RNA-seq data and qRT-PCR in all selected genes, whether in wild type or mutants (R = 0.82 ~ 0.99; Supplementary Figure S3).

### Functional Annotation of Key DEGs in Stalk Development Stages of Fruit Spines

**Figure 8:**
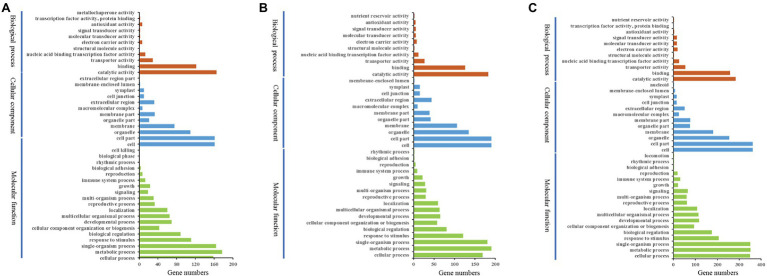
Histogram presentation of gene ontology (GO) classifications. Left *y*-axis indicates the percentage of DEGs in the subcategories of each main category. Right y-axis indicates the number of DEGs in each subcategory. *x*-axis indicates the GO subcategories. **(A)** DEG-IIs of wild type. **(B)** All DEG-II–IIIs of wild type. **(C)** DEG-IVs of wild type.

There were 389 DEGs with the highest expression levels at stage II in wild type (for convenience, we called them DEG-IIs). To evaluate the functional categories of the DEG-IIs, we compared these DEGs against the Gene Ontology (GO) and Kyoto Encyclopedia of Genes and Genomes (KEGG) databases (Figure 8A). At the same time, we also performed in-depth tracking with a GO acyclic graph to analyze the GO categories in which the DEG-IIs were enriched (Supplementary Figure S4). The GO categorization of DEG-IIs according to biological processes, molecular functions, and cellular components was analyzed. Categories based on biological processes demonstrated that the DEG-IIs were mainly enriched in lipid metabolic processes, amino acids, iron transport, signal transduction, and anatomical structure development and morphogenesis. When we classified the DEG-IIs according to cellular components, we found that the DEG-IIs were mainly involved in plasma membrane, cell wall, and intracellular organelle. In the molecular function category, oxidoreductase activity, transferase activity, and hydrolase activity were the subgroups with the highest numbers of DEG-IIs. We also mapped these DEG-IIs to the KEGG database to identify pathways in which DEG-IIs might be involved. The DEG-IIs were mainly enriched in amino acid metabolism, linoleic acid metabolism, and plant–pathogen interaction (Supplementary Figure S5; Supplementary Table S2). To date, a variety of phytohormones have been shown to be involved in the initiation and branching of plant trichomes (Yang and Ye, 2013; Yang et al., 2015). We also found that the DEG-IIs were involved in signal transduction and response for almost all kinds of phytohormones (Supplementary Table S3), including auxin, ethylene (ET), abscisic acid (ABA), gibberellin (GA), cytokinin (CK), brassinolide (BR), jasmonic acid (JA), and salicylic acid (SA). Specifically, auxin polar transport was the category involving the most genes (19 genes). In addition, we found that most of the DEG-IIs related to phytohormones were also involved in tissue pattern formation (Supplementary Table S4). Previous studies have also reported that genes involved in meristem regulation may play an important role in the development of spines (Chen et al., 2014). Among the DEG-IIs, we identified 30 genes involved in meristem regulation and cell cycle (Supplementary Table S4). Some of their homologs have also been shown to participate in the polarity development of tissues. For example, *AGO10* (the homolog in cucumber, *Csa3G144740*) regulates leaf polarity establishment by repressing miR165 and miR166 in *Arabidopsis* (Liu et al., 2009). *ATHB-14* (the homolog in cucumber, *Csa6G525430*) is involved in the fate regulation of adaxial–abaxial polarity in the ovule primordium in *Arabidopsis* (McConnell et al., 2001). Transcription factors (TFs) are the key nodes of gene regulatory networks, and expression level changes of transcription factors will affect the expression levels of many downstream genes. We identified 54 transcription factors among the DEG-IIs (Supplementary Table S5). The 54 TFs could be subdivided into 20 categories according to their transcription factor family. The most common category was the HD-ZIP transcription factor family, which contains six genes. Some homologs of TFs in other plants have been identified to be involved in the initiation and development of tissues. For example, the homolog of *Csa7G428260*, encoding a C2H2 transcription factor, regulates the normal pattern of cell division, expansion, and differentiation during morphogenesis of the silique in *Arabidopsis* (Marsch-Martínez et al., 2014). MYB21, a homolog of *Csa3G168940*, regulates stamen filament elongation in the late developed flowers in *Arabidopsis* (Shin et al., 2002).

In addition, 414 genes showed no significant difference in expression level between stage II and stage III but were significantly downregulated at stage IV (for convenience, we called them DEG-II–IIIs), indicating that these genes may play a role in maintaining the development of the stalk. In the GO category biological process, DEG-II–IIIs were mainly enriched in lipid metabolic processes, amino acids, ion transport, signal transduction, multicellular organismal development, and response to endogenous hormone (Figure 8B; Supplementary Figure S6). When classifying the DEG-II–IIIs according to GO cellular components, we found that DEG-II–IIIs were mainly involved in plasma membranes, cell walls, and intracellular organelles. Compared to the DEG-IIs, more DEG-II–IIIs were associated with the cytoskeleton. Analysis of the GO category molecular function demonstrated that oxidoreductase activity, transferase activity, hydrolase activity, and lyase activity were subgroups with the highest number of DEG-II–IIIs. The results of KEGG analysis for DEG-II–IIIs indicated that the biosynthesis pathways of amino acid metabolism, linoleic acid metabolism, and phenylpropanoid biosynthesis were significantly enriched for the DEG-II–IIIs (Supplementary Table S2; Supplementary Figure S5). The DEG-II–IIIs included 36 TFs (Supplementary Table S5), and these TFs belonged to 20 transcription factor families. HD-ZIP was still the family with the largest number of enriched genes, indicating that HD-ZIP transcription factors play an important role in the development of cucumber spines. For example, the homologs of Csa1G031750, Csa1G064670, HDG11, and ATML1 are well-known TFs that can affect the development of trichome branching in *Arabidopsis* (Szymanski and Marks, 1998). There were also genes associated with plant hormones found among the DEG-II–IIIs, while compared with the DEG-IIS, these genes were involved not only in polar transport of hormones (Supplementary Table S3) but also in the biosynthesis and transport of substances, as well as stress resistance. A few genes that regulate the development of fruit trichomes by interacting with the cytoskeleton were also found among the DEG-II–IIIs. For example, KIC (encoded by *Csa6G106800*) can interact with kinesin motor protein in a calcium-dependent manner to regulate the pattern of trichome branching in *Arabidopsis* (Reddy et al., 2004). The expression level of *Csa6G106800* was downregulated 1.6-fold in stage IV compared with stages II and III.

We found only 26 genes whose expression levels were significantly higher in stage III than in stage II and stage IV (for convenience, we called them DEG-IIIs; Supplementary Table S1). According to GO analysis and KEGG analysis, these 26 genes were mainly related to secondary metabolism and protein processing and transportation. At the same time, we did not find any genes related to hormones or meristems among the DEG-IIIs.

These results indicate that the stalk development of fruit spines depends on the polar transport of phytohormones regulated by the cytoskeleton. This developmental process is continuous, and most genes reach their peak expression levels at the initial developmental stage of fruit spines.

### Functional Annotation of Key DEGs in the Base Developmental Stages of Fruit Spines

Stage IV was the key period for the base development of fruit spines, and the pattern of cell division and proliferation was different from those of other stages, implying a big change in the involved gene regulatory network. There were 722 genes with the highest expression levels in stage IV (for convenience, we called them DEG-IVs; Supplementary Table S1). The results of GO and KEGG analyses also showed that the function of DEG-IVs was different from those of DEG-IIs and DEG-IVs.

In the biological process category, DEG-IVs were enriched in single-organism processes, cellular processes, and metabolic processes (Figure 8C). The GO directed acyclic graph showed clearer details of DEG-IV enrichment (Supplementary Figure S7). Signal transduction, multicellular organism, transport, anatomical structure, lipid metabolism, and amino acid metabolism were the most common biological processes in which DEG-IVs were involved. When we classified the DEG-IVs according to cellular components, we found that in addition to being enriched in plasma membranes and cell walls, DEG-IVs were also enriched in protein complexes and intracellular membranes. Furthermore, compared with DEG-IIs and DEG-II–IIIs, DEG-IVs also had more genes related to the cytoskeleton. For molecular function, unlike the DEG-IIs and DEG-II–IIIs, which were only enriched in catalytic activity (oxidoreductase activity, transferase activity, hydrolase activity, and lyase activity), the DEG-IVs also included some genes enriched in transporter activity. The results of KEGG analysis of the DEG-IVs indicated that the biosynthesis pathways of carbon metabolism, glyoxylate and dicarboxylate metabolism, and glycine, serine, and threonine metabolism were significantly enriched (Supplementary Table S2; Supplementary Figure S5). Another difference from the stalk developmental stage was that bHLH was the transcription factor family with the most genes among the DEG-IVs, with a total of 13 genes (Supplementary Table S5). Although none of these bHLHs have been shown to be involved in the development of plant trichomes, several bHLH transcription factors have been shown to be involved in the specialization of epidermal cells in *Arabidopsis*. For instance, SCRM (the homolog in cucumber, *Csa1G051760*) mediates stomatal differentiation in the epidermis by controlling MUTE and FAMA in *Arabidopsis* (Ohashi-Ito and Bergmann, 2006; Pillitteri et al., 2007; De Marcos et al., 2017). Coincidentally, the homologous genes of *FAMA*, *MUTE*, *Csa3G150010*, and *Csa3G131940* were also present among the DEG-IVs, suggesting that this set of bHLH complexes may be involved in the development of cucumber trichomes. Similar to the results for the DEG-IIs and DEG-II–IIIs, we also identified multiple phytohormones related to the DEG-IVs. We found a total of 71 genes involved in the response of eight phytohormones (Supplementary Table S3). Among them, auxin response-related genes were still the most abundant (20 genes), indicating the important role of auxin in the development of cucumber trichomes. In addition, there were 50 genes involved in the meristem and cell cycle among the DEG-IVs (Supplementary Table S4). However, we did not find any genes among the DEG-IVs that are known to be involved in trichome development in *Arabidopsis*, suggesting that the base is a unique structure of cucumber trichomes and the development mechanism may be very different from that of unicellular trichomes.

### Auxin Polar Transport and Cytoskeletal Signaling Are the Main Factors Causing Phenotypic Differences Between the Wild Type and Mutant

Stage III was the critical period for the difference between the wild type and mutant. In stage III, the development of cells that made up the stalk was disrupted and incomplete in the mutant (Figure 4), indicating that the normal gene regulatory network was disturbed, so we focused on DEGs that were not differentially expressed at stage II between the wild type and mutant but were significantly differentially expressed at stage III. There were 628 genes that showed this expression pattern in the transcriptome data (Supplementary Table S1).

Then, we performed GO and KEGG analyses to evaluate the functions of these DEGs. In the biological process category, upregulated genes in mutant were more enriched in the processes of multicellular organismal development and anatomical structure development (Supplementary Figure S8), while downregulated genes in mutant were more enriched in the processes of endogenous hormone response and amino acid metabolism (Supplementary Figure S9). When classifying the DEGs according to molecular function, we found that the downregulated genes were enriched not only in the activities of oxidoreductase, transferase, and hydrolase, similar to upregulated genes, but also in lyase activity. The analysis of cellular components demonstrated that the enriched processes of upregulated genes and downregulated genes were roughly the same, except that the upregulated genes were also enriched in the processes of intracellular membrane-bound organelle and plasma membrane. The results of KEGG analysis showed that the upregulated and downregulated genes participated in different pathways (Supplementary Figures S10, S11). Pentose and glucuronate interconversions, phenylalanine metabolism, and tryptophan metabolism were significantly enriched for upregulated genes. Downregulated genes were mainly enriched in glutathione metabolism, plant–pathogen interaction, and alpha-linolenic acid metabolism.

By further exploring the functions of DEGs, we found that auxin polar transport was associated with the upregulated genes in mutant. A series of auxin carrier proteins, namely, LAX4 (*Csa4G308640*), LAX5 (*Csa7G010800*), PID2 (*Csa2G006100*), and PIN1 (*Csa4G430820*), were detected among the upregulated genes. NPY1 (the homolog in cucumber, *Csa2G382680*) can regulate cotyledon development through the control of PIN1 polarity in *Arabidopsis* (Cheng et al., 2008), and *Csa2G382680* was also found among the upregulated genes. Among the downregulated genes in mutant, there were far more genes related to the meristem and cell cycle than there were among the upregulated genes. Among those downregulated genes, genes related to calcium signaling in the cytoskeleton were very noteworthy. A series of calcium sensors, such as CML46, CML38, and CML11, were found among the downregulated genes. PCM1 (*Csa3G167380*), a kind of calmodulin that mediates the control of many protein kinases, ion channels, and other proteins by calcium signaling, was also detected among the downregulated genes. Furthermore, genes that directly affect the cell cycle by interacting with the cytoskeleton were also detected among the downregulated genes. KIC (the homolog in cucumber, *Csa6G106800*) is a calmodulin that can interact with kinesin in the cytoskeleton to directly affect the branch number of trichomes in *Arabidopsis* (Reddy et al., 2004). RANGAP1 (the homolog in cucumber, *Csa2G355000*) is an activator of the cell polarity molecule Ran GTPase and plays a role in spatial signaling during cell division (Xu et al., 2008).

### CsTs Can Interact With CsVTI11, a Key Factor Involved in Auxin Signal Transmission

**Figure 9:**
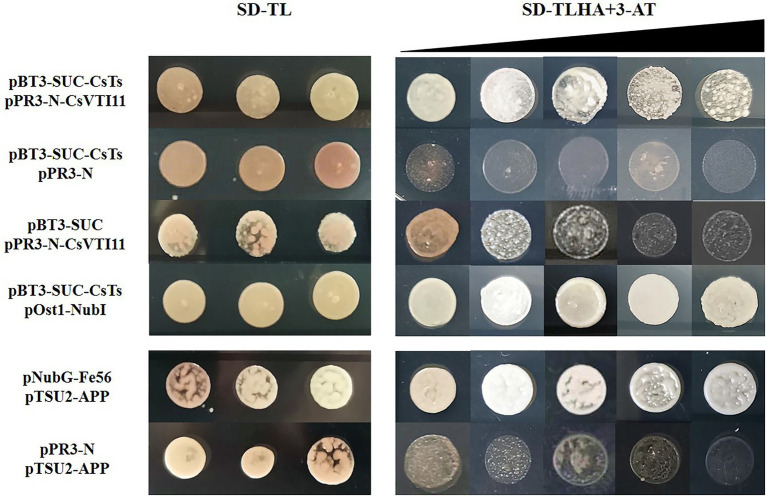
Yeast two-hybrid analysis. pBT3-SUC-CsTs+pPR3-N-CsVTI11: intermolecular interaction between CsVTI11 and CsTs; pBT3-SUC-CsTs+pPR3-N and pBT3-SUC + pPR3-N-CsVTI11: self-activation test of plasmids; pBT3-SUC-CsTs+pOst1-NubI: cytotoxicity test of plasmids; pNubG-Fe65 + pTSU2-APP: positive control group; and pPR3-N + pTSU2-APP: negative control group. The concentration of 3-AT is 0, 1, 2.5, 5, and 7.5 mM, respectively.

**Figure 10:**
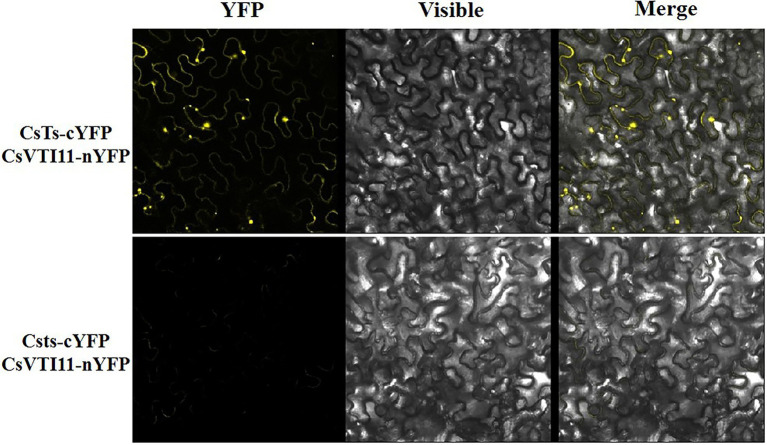
BiFC analysis between CsVTI11 and CsTs or Csts. BiFC analysis of the physical interaction between CsVTI11 (fused with the C-terminal fragment of YFP) and CsTs (fused with the N-terminal fragment of YFP) or Csts (fused with the N-terminal fragment of YFP). Different combinations of the fused constructs were co-expressed in leaves of *N. benthamiana*, and the cells were then visualized using confocal microscopy.

In plant cells, the cytoskeleton system can affect auxin polar distribution by regulating the efficiency of auxin transport, and auxin also dynamically regulates the organization pattern of the cytoskeleton system. This feedback regulation between them is mediated by vesicle-associated proteins (Zou et al., 2019). Vacuolar SNARE VTI11/ZIG1 is a protein involved in auxin signal transmission in plants, and it can regulate cell size and division in an interdependent manner with auxin (Yano et al., 2003; Löfke et al., 2015). *CsVTI11* is a homolog of *AtVTI11* (identity of protein is about 76%) and had the same expression trend as *CsTs* in different development stages of fruit spines. Through experimenting with Y2H (Figure 9; Supplementary Figure S12) and BIFC (Figure 10), we found that *CsTs* can interact with *CsVTI11* (the protein homology with AtVTI11 was 76%) *in vivo*, but there is no such interaction relationship between the *Csts* and *CsVTI11*. This result is consistent with the transcriptome analysis to some extent, indicating that *CsTs* may be involved in the polar transport of auxin in the cells during the development of fruit spines.

## Discussion

Benefitting from the fact that the cells that make up the fruit spines were larger than those on other tissues and existed on the surface of the fruit at a low density, we can more easily observe the development of spine morphology in detail by microscopy. Therefore, spines provide an excellent model for studying cell differentiation and proliferation. Due to the lack of a variety of representative materials, although some important transcription factors have been reported, previous research on the development of fruit spines always focused on the presence (Wang et al., 2016), density (Yang et al., 2018), and size (Li et al., 2015; Chen et al., 2016) of fruit spines. There has been no precise division of the potential stages during the development of fruit spines, and an in-depth understanding of the developmental patterns of the stalk and base of fruit spines is lacking.

Fortunately, we recently obtained a natural mutant in which the spine cells have an abnormal division pattern, making it possible to divide the important stages in the development of fruit spines. In this study, we divided the development of fruit spines into stages I to IV (Figure 2). Stage I mainly involved the specific development of some epidermal cells to initiate the formation of fruit spines. At stage II, the stalk of spines began to develop according to a single-cell relay, and the mark of the end of this stage was the full extension of the fourth single cell of the stalk. In stage III, the stalk continued to develop, accompanied by the expansion of the ovary, an increase in the rigidity of the fruit spines, and eventually the end of the development of the stalk (7 ~ 8 cells). Stage IV mainly involved the development of the base of spines, and the cell population that made up the base kept dividing and expanding to eventually form straight and hard fruit spines. Comparing the wild type with the mutant (Figures 2, 4), we found that stage III was the key stage in determining whether the development of fruit spines was abnormal. The wild-type fruit spines will develop a complete stalk at this stage, but the stalk in the mutant stage III is no longer fully extended, the single cells of the stalk are flat, and some even divide into multiple cells, which precludes mutant stage IV, and the difference between the stalk and base is blurred.

The development of multicellular trichomes is a process involving rapid cell proliferation and division, and cell proliferation requires amino acids and lipids to provide nutrition (Nurse and Wiemken, 1974; Natter and Kohlwein, 2013). Previous studies have shown that genes related to lipid and amino acid metabolism were enriched in the process of trichome development in tobacco (Yang et al., 2015). In this study, regardless of the fruit spine developmental stage, genes related to amino acid and lipid metabolism were significantly upregulated. This indicates that despite differences in the structure of trichomes between different plants, amino acid and lipid metabolism play a very important role in the development of multicellular trichomes.

To date, a variety of phytohormones have been shown to be involved in the initiation and branching of trichomes (Yang et al., 2014, 2015). For example, in *Arabidopsis*, GA and JA are synergistically associated with the induction of trichomes, but SA antagonizes the induction by GA of trichome density and number (Traw and Bergelson, 2003). Ethylene can affect trichome branching (Traw and Bergelson, 2003). The application of CK causes trichome formation on the inflorescence stem (Yu et al., 2010). In tomato and tobacco, JA, ET, and auxin play an important role in the development of multicellular trichomes (Yang et al., 2015) The expression of many genes related to diverse phytohormone signaling pathways changed in our study, including auxin, BR, ABA, GA, ET, JA, SA, and CK. Among the upregulated DEGs at different fruit spine developmental stages, the number of genes related to the auxin response was the largest, especially in stage II and stage III, and the enrichment of auxin polar transport-related genes was significant. The unique ability of auxin to move in a polar fashion allows for differential tissue distribution, which is a key factor in tropic growth, apical dominance, lateral root initiation, vascular development, and embryo patterning (Vanneste and Friml, 2009). Auxin polar transport-related genes were significantly upregulated at stage II and stage III, indicating that cell polarity played a dominant role in the initiation of cucumber trichomes and the maintenance of early stage stalk structural development. In addition, only a few GA response-related DEGs were detected at each stage. Combined with research on tobacco trichomes (Yang et al., 2015), this further illustrates that GA may play a minor role in the development of multicellular trichomes. Previous studies did not find that ABA was involved in the development and regulation of plant trichomes, but we detected some upregulated DEGs related to the ABA response in different stages of fruit spine development, especially in stage IV. In this stage, a total of 18 ABA response-related genes were detected, indicating that ABA may participate in the development of the base structure of multicellular trichomes.

In recent years, many key regulators and their downstream genes participating in the regulation of trichome formation have been identified (Oppenheimer et al., 1991; Payne et al., 2000; Zhang et al., 2003; Ishida et al., 2007; Yang et al., 2015). In our study, we detected that the expression levels of a large number of TFs were changed during fruit spine development. These transcription factors were from 45 gene families, indicating that different stages of fruit spine development involve the adjustment of gene regulatory networks. In stage II and stage III, we found that HD-Zip transcription factors were the most active family involved in the development of fruit spines, indicating that HD-ZIP transcription factors mainly regulate the initial development of trichomes and stalk structure. However, at stage IV, bHLH was the most involved transcription factor family, suggesting that the multicellular base structure was mainly regulated by bHLH transcription factors.

The mutant (*Csts*) used in this study was initially defined as having a type of tender fruit spine (Guo et al., 2018). With continuous tracking of the developmental stage of the fruit spines, we found that the most direct reason for the fruit spines being more easily bent was their developmental deformity in the mutant, which made the combination of the base and epidermis unstable (Figure 4). Differences in fruit spines between the wild type and mutant were found in stage III. Starting from the fifth single cell of the stalk, all single cells that made up the stalk failed to become fully elongated, and then, the single cells that made up the stalk began to split into a multicellular structure similar to the base (Figure 4). Therefore, *CsTs* should play a role in cell division and differentiation patterns in the development of fruit spines. *CsTs* encodes a C-type lectin receptor-like kinase. *C-type LecRLK* is a mysterious gene. Most plants have only one or two of this type of receptor-like kinase (Scita et al., 1999; Shumayla et al., 2016; Yang et al., 2016). Previous studies on model plants did not clarify its specific biological functions. This suggests that *CsTs* may be involved in a genetic network that we still do not fully understand. Fruit spines are specialized from cucumber epidermal cells and contain several different structures (top, stalk, and base), the development procession of which must involve precise regulation between the auxin and cytoskeleton system. In our transcriptome data, although no matter in the mutant or the wild type, the expression level of *CsTs* (*Csa1G056960*) did not change significantly during the three developmental stages of the fruit spines. However, by analyzing the function of DEGs between wild type and mutant, some valuable findings were made. There were 628 genes with no expression difference in stage II but a significant expression difference in stage III between the wild type and mutant. In the mutant, the expression levels of a series of calcium sensors were downregulated. Calcium is an important signaling molecule in the cytoskeleton and has important functions in regulating auxin transport (Lee et al., 1984; Vidali et al., 2001; Toyota et al., 2008). Correspondingly, upregulated genes in the mutant were mainly related to the polar transport of auxin. A previous study on zucchini showed that tyrosine kinase can regulate the auxin efflux carrier by phosphorylation (Bernasconi, 2010). The dynamic feedback regulation between the cytoskeleton system and auxin polarity distribution is achieved through the regulation of vesicle-associated proteins (Zou et al., 2019). Coincidentally, we found a key factor involved in auxin signal transmission, CsVTI11, can interact with CsTs *in vivo*, but this interaction does not exist between CsVTI11 and Csts. Based on these results, we speculate that CsTs, as one type of receptor protein kinase, is responsible for receiving intercellular signals and then sends signals to vesicle-associated proteins to exercise fine control over the cytoskeletal system and auxin polarity distribution and ensure the correct development of different cell types in fruit spines. In *ts*, Csts will lead to the failure of transmission of intercellular signals to downstream proteins, resulting in the abnormal expression of the cytoskeletal system and auxin transporter-related genes and causing the metamorphosis of fruit spines in *ts*. Similar results have been found in tomatoes. SlHDZIV8 is an HD-Zip IV transcription factor of tomato and it can affect the development of trichomes by regulating actin filament distribution (Xie et al., 2020). The RNAi of SlHDZIV8 results in shorter stalk cells (Xie et al., 2020), a phenotype that resembles the stalk cell of *ts* mutant at stage III. These findings not only confirm the influence of HD-ZIP transcription factors on the development of multicellular trichomes, but also indicates the importance of the correct arrangement of the cell cytoskeleton for the growth of multicellular trichomes.

## Data Availability Statement

The original contributions presented in the study are publicly available. This data can be found at National Center for Biotechnology Information (NCBI) BioProject database under accession number PRJNA660492 (https://www.ncbi.nlm.nih.gov/bioproject/PRJNA660492).

## Author Contributions

DL contributed to the bioinformatics analysis and writing of the manuscript. QZ helped with experiment of BIFC and Y2H. P-CQ and YY helped with the RNA extraction and qRT-PCR. H-LH, J-XS, and K-YZ helped with growing plants. J-AP contributed to plant materials. RC, GW, and J-SP provided critical insights and revised the manuscript. All authors read and approved the final manuscript.

## Funding

This study was supported by the Project of Science and Technology Commission of Shanghai Municipality (no. 18391900300), the Agri-X Project of Shanghai Jiao Tong University (Agri-X2017011), and the National Natural Science Foundation of China (no. 31672148).

## Conflict of Interest

J-AP was employed by Tianjin Derit Seeds Company Limited, Tianjin, China.

The remaining authors declare that the research was conducted in the absence of any commercial or financial relationships that could be construed as a potential conflict of interest.

## Publisher’s Note

All claims expressed in this article are solely those of the authors and do not necessarily represent those of their affiliated organizations, or those of the publisher, the editors and the reviewers. Any product that may be evaluated in this article, or claim that may be made by its manufacturer, is not guaranteed or endorsed by the publisher.

## Supplementary Material

The Supplementary Material for this article can be found online at: https://www.frontiersin.org/articles/10.3389/fpls.2022.797433/full#supplementary-material

Supplementary Table S1 The DEGs in this study. DEG-IIs represent differentially expressed genes (DEGs) with the highest expression levels at stage II of wild type; DEG-II–IIIs represent differentially expressed genes (DEGs) that showed no significant difference in expression level between stage II and stage III but were significantly downregulated at stage IV (for convenience, we called them DEG-II–IIIs) of wild type; DEG-IVs represent differentially expressed genes (DEGs) with the highest expression levels at stage IV of wild type; u-regulation represents upregulated differentially expressed genes (DEGs) in mutant; and downregulation represents downregulated differentially expressed genes (DEGs) in mutant.

Supplementary Table S2 KEGG analysis of all DEGs. DEG-IIs represent differentially expressed genes (DEGs) with the highest expression levels at stage II of wild type; DEG-II–IIIs represent differentially expressed genes (DEGs) that showed no significant difference in expression level between stage II and stage III but were significantly downregulated at stage IV (for convenience, we called them DEG-II–IIIs) of wild type; DEG-IVs represent differentially expressed genes (DEGs) with the highest expression levels at stage IV of wild type; upregulation represents upregulated differentially expressed genes (DEGs) in mutant; and downregulation represents downregulated differentially expressed genes (DEGs) in mutant.

Supplementary Table S3 All DEGs related to phytohormones. DEG-IIs represent differentially expressed genes (DEGs) with the highest expression levels at stage II of wild type; DEG-II–IIIs represent differentially expressed genes (DEGs) that showed no significant difference in expression level between stage II and stage III but were significantly downregulated at stage IV (for convenience, we called them DEG-II–IIIs) of wild type; DEG-IVs represent differentially expressed genes (DEGs) with the highest expression levels at stage IV of wild type; upregulation represents upregulated differentially expressed genes (DEGs) in mutant; and downregulation represents downregulated differentially expressed genes (DEGs) in mutant. FC, fold change; FPKM, fragments per kilobase of transcript per million fragments mapped.

Supplementary Table S4 All DEGs related to meristem and cell cycle. DEG-IIs represent differentially expressed genes (DEGs) with the highest expression levels at stage II of wild type; DEG-II–IIIs represent differentially expressed genes (DEGs) that showed no significant difference in expression level between stage II and stage III but were significantly downregulated at stage IV (for convenience, we called them DEG-II–IIIs) of wild type; DEG-IVs represent differentially expressed genes (DEGs) with the highest expression levels at stage IV of wild type; upregulation represents upregulated differentially expressed genes (DEGs) in mutant; and downregulation represents downregulated differentially expressed genes (DEGs) in mutant. FC, fold change; FPKM, fragments per kilobase of transcript per million fragments mapped.

Supplementary Table S5 Transcription factors in DEGs. DEG-IIs represent differentially expressed genes (DEGs) with the highest expression levels at stage II of wild type; DEG-II–IIIs represent differentially expressed genes (DEGs) that showed no significant difference in expression level between stage II and stage III but were significantly downregulated at stage IV (for convenience, we called them DEG-II–IIIs) of wild type; DEG-IVs represent differentially expressed genes (DEGs) with the highest expression levels at stage IV of wild type; upregulation represents upregulated differentially expressed genes (DEGs) in mutant; and downregulation represents downregulated differentially expressed genes (DEGs) in mutant. FC, fold change; FPKM, fragments per kilobase of transcript per million fragments mapped.

Supplementary Table S6 Primers for qRT-PCR in this study.

Supplementary Figure S1 Correlation coefficients for every two samples. Heat map color represents the correlation coefficient; the numbers in the squares represent correlations between two samples, the closer the color of a square is to light cyan, and the higher the correlation, the closer the color of a square is to magenta, the lower the correlation. **(A)** Wild type; **(B)** Mutant (*Csts*).

Supplementary Figure S2 qRT-PCR analysis of *CsTs* and *Csts*. The red line CsTs-Q represents the expression levels analyzed by qRT-PCR of *CsTs* in different development stages of fruit spines. The blue line Csts-Q represents the expression levels analyzed by qRT-PCR of *Csts* in different development stages of fruit spines. CsTs-Q and Csts-Q represent value of FPKM (fragments per kilobase per million) in different development stages of fruit spines.

Supplementary Figure S3 qRT-PCR analysis of selected genes. The blue line represents the value of FPKM (fragments per kilobase per million) and the red line represents the expression levels analyzed by qRT-PCR; the uppercase genes on the left row belong to wild type; the lower case genes on the right row belong to mutant; and Pearson correlation coefficient had an R range between 0.82 and 0.99 and averaged 0.979.

Supplementary Figure S4 GO acyclic graph of DEG-IIs. DEG-IIs represent differentially expressed genes (DEGs) with the highest expression levels at stage II of wild type.

Supplementary Figure S5 KEGG enrichment of differentially expressed genes in different developmental stage of cucumber fruit spines. **(A)** KEGG enrichment of DEG-IIs. DEG-IIs represent differentially expressed genes (DEGs) with the highest expression levels at stage II of wild-type fruit spines. **(B)** KEGG enrichment of DEG-II–IIIs. DEG-II–IIIs represent differentially expressed genes (DEGs) that showed no significant difference in expression level between stage II and stage III but were significantly downregulated at stage IV (for convenience, we called them DEG-II–IIIs) of wild-type fruit spines. **(C)** KEGG enrichment of DEG-IVs. DEG-IVs represent differentially expressed genes (DEGs) with the highest expression levels in stage IV of wild-type fruit spines.

Supplementary Figure S6 GO acyclic graph of DEG-II–IIIs. DEG-II–IIIs represent differentially expressed genes (DEGs) that showed no significant difference in expression level between stage II and stage III but were significantly downregulated at stage IV (for convenience, we called them DEG-II–IIIs) of wild type.

Supplementary Figure S7 GO acyclic graph of DEG-IVs. DEG-IVs represent differentially expressed genes (DEGs) with the highest expression levels in stage IV of wild type.

Supplementary Figure S8 GO acyclic graph of upregulated genes in mutant.

Supplementary Figure S9 GO acyclic graph of downregulated genes in mutant.

Supplementary Figure S10 KEGG enrichment of upregulated genes in mutant.

Supplementary Figure S11 KEGG enrichment of downregulated genes in mutant.

Supplementary Figure S12 The original experimental pictures of Yeast two-hybrid analysis, including four biological repeats of each combination. pBT3-SUC-CsTs + pPR3-N-CsVTI11: intermolecular interaction between CsVTI11 and CsTs; pBT3-SUC-CsTs + pPR3-N and pBT3-SUC + pPR3-N-CsVTI11: self-activation test of plasmids; pBT3-SUC-CsTs + pOst1-NubI: cytotoxicity test of plasmids; pNubG-Fe65 + pTSU2-APP: positive control group; and pPR3-N + pTSU2-APP: negative control group. The concentration of 3-AT is 0, 1, 2.5, 5, and 7.5 mM, respectively. SD-TL and SD-TLHA represent different deficient selective medium, and TLHA represents Leucine, Tryptophan, Histidine, and Adenine.

## Glossary

**Table:** 

Term	Definitions
Wt	Wild type
Mu	Mutant
DEGs	Differential expression genes
FPKM	Fragments per kilobase of transcript per million fragments mapped
GL1	GLABRA1
TTG1	TRANSPARENT TESTA GLABRA1
bHLH	basic helix–loop–helix
GL3	GLABRA3
EGL3	ENHANCER OF GLABRA3
TRY	TRIPTYCHON
CPC	CAPRICE
ETC	ENHANCER OF TRY AND CPC
SEM	Scanning electron microscope
Tril	Trichome-less
Tu	Tuberculate fruit
Ts	Tender spine
LecRLK	Lectin receptor-like kinase
Mict	Micro-trichome
GO	Gene Ontology
KEGG	Kyoto Encyclopedia of Genes and Genomes
COG	Clusters of Orthologous Groups
NR	Nonredundant
TF	Transcription factor
HD-ZIP	Homeodomain-leucine zipper
DEG-IIs	DEGs with the highest expression levels at stage II in wild type
DEG-II–IIIs	DEGs showed no significant difference in expression level between stage II and stage III but were significantly downregulated at stage IV in wild type
DEG-IIIs	DEGs with the highest expression levels at stage III in wild type
DEG-IVs	DEGs with the highest expression levels at stage IV in wild type
ET	Ethylene
ABA	Abscisic acid
GA	Gibberellin
CK	Cytokinin
BR	Brassinolide
JA	Jasmonic acid
SA	Salicylic acid

## Footnotes

^1^ https://www.ncbi.nlm.nih.gov/bioproject/PRJNA660492

^2^ http://ccb.jhu.edu/software/hisat2/index.shtml

^3^ http://web.expasy.org/docs/relnotes/relstat.html

^4^ http://pfam.xfam.org/

^5^ http://www.bioconductor.org/packages/2.11/bioc/html/topGO.html

^6^ http://cucurbitgenomics.org/
