# Supplementary material for: Comparative Transcriptome Analysis of Hard and Tender Fruit Spines of Cucumber to Identify Genes Involved in the Morphological Development of Fruit Spines
Source: Front Plant Sci. 2022 Mar 15;13:797433. doi: 10.3389/fpls.2022.797433 (PMC8965156; doi:10.3389/fpls.2022.797433)
Supplement: Supplementary Table S1 — The DEGs in this study. DEG-IIs represent differentially expressed genes (DEGs) with the highest expression levels at stage II of wild type; DEG-II–IIIs represent differentially expressed genes (DEGs) that showed no significant difference in expression level between stage II and stage III but were significantly downregulated at stage IV (for convenience, we called them DEG-II–IIIs) of wild type; DEG-IVs represent differentially expressed genes (DEGs) with the highest expression levels at stage IV of wild type; u-regulation represents upregulated differentially expressed genes (DEGs) in mutant; and downregulation represents downregulated differentially expressed genes (DEGs) in mutant. [file Data_Sheet_1.ZIP › Additional files/Figure S5.pdf]

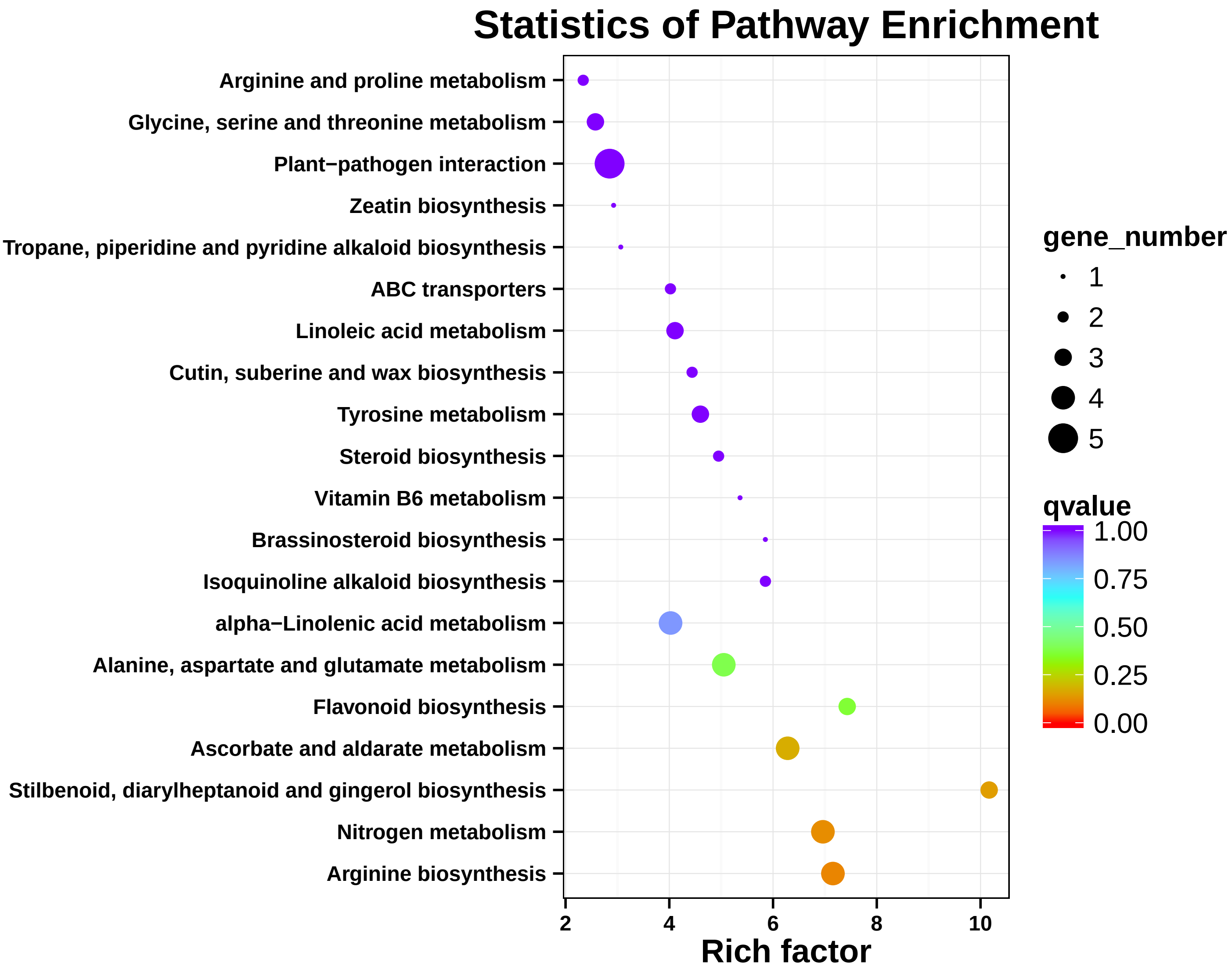

Statistics of Pathway Enrichment
Arginine and proline metabolism
Glycine, serine and threonine metabolism
Plant−pathogen interaction
Zeatin biosynthesis
Tropane, piperidine and pyridine alkaloid biosynthesis
ABC transporters
Linoleic acid metabolism
Cutin, suberine and wax biosynthesis
Tyrosine metabolism
Steroid biosynthesis
Vitamin B6 metabolism
Brassinosteroid biosynthesis
Isoquinoline alkaloid biosynthesis
alpha−Linolenic acid metabolism
Alanine, aspartate and glutamate metabolism
Flavonoid biosynthesis
Ascorbate and aldarate metabolism
Stilbenoid, diarylheptanoid and gingerol biosynthesis
Nitrogen metabolism
Arginine biosynthesis
2
4
6
8
10
Rich factor
gene_number
1
2
3
4
5
qvalue
1.00
0.75
0.50
0.25
0.00

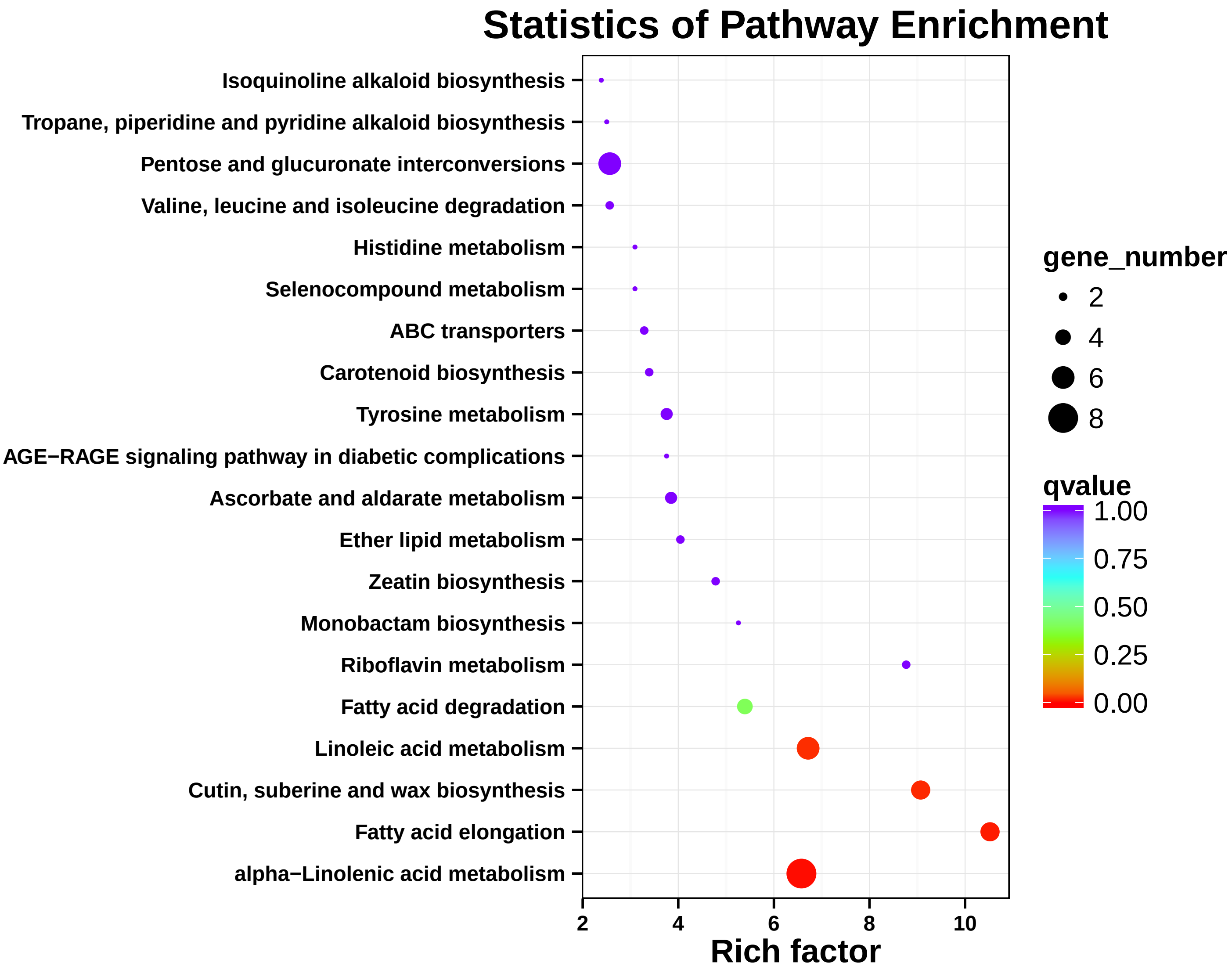

Statistics of Pathway Enrichment
Isoquinoline alkaloid biosynthesis
Tropane, piperidine and pyridine alkaloid biosynthesis
Pentose and glucuronate interconversions
Valine, leucine and isoleucine degradation
Histidine metabolism
Selenocompound metabolism
ABC transporters
Carotenoid biosynthesis
Tyrosine metabolism
AGE−RAGE signaling pathway in diabetic complications
Ascorbate and aldarate metabolism
Ether lipid metabolism
Zeatin biosynthesis
Monobactam biosynthesis
Riboflavin metabolism
Fatty acid degradation
Linoleic acid metabolism
Cutin, suberine and wax biosynthesis
Fatty acid elongation
alpha−Linolenic acid metabolism
2
4
6
8
10
Rich factor
gene_number
2
4
6
8
qvalue
1.00
0.75
0.50
0.25
0.00

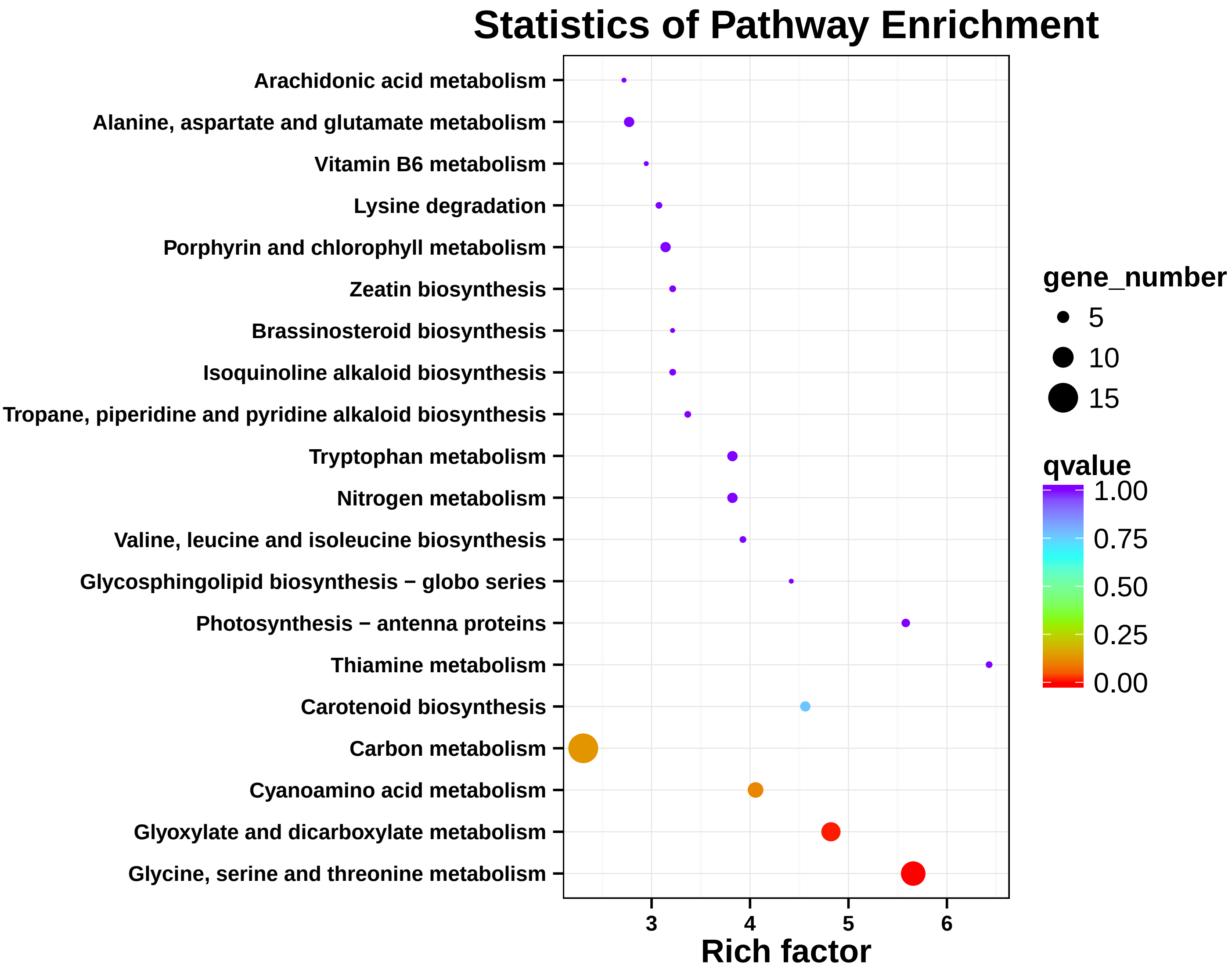

Statistics of Pathway Enrichment
Arachidonic acid metabolism
Alanine, aspartate and glutamate metabolism
Vitamin B6 metabolism
Lysine degradation
Porphyrin and chlorophyll metabolism
Zeatin biosynthesis
Brassinosteroid biosynthesis
Isoquinoline alkaloid biosynthesis
Tropane, piperidine and pyridine alkaloid biosynthesis
Tryptophan metabolism
Nitrogen metabolism
Valine, leucine and isoleucine biosynthesis
Glycosphingolipid biosynthesis − globo series
Photosynthesis − antenna proteins
Thiamine metabolism
Carotenoid biosynthesis
Carbon metabolism
Cyanoamino acid metabolism
Glyoxylate and dicarboxylate metabolism
Glycine, serine and threonine metabolism
3
4
5
6
Rich factor
gene_number
5
10
15
qvalue
1.00
0.75
0.50
0.25
0.00
