# Supplementary figures and images for: Comparative Transcriptome Analysis of Hard and Tender Fruit Spines of Cucumber to Identify Genes Involved in the Morphological Development of Fruit Spines
Source: Front Plant Sci. 2022 Mar 15;13:797433. doi: 10.3389/fpls.2022.797433 (PMC8965156; doi:10.3389/fpls.2022.797433)

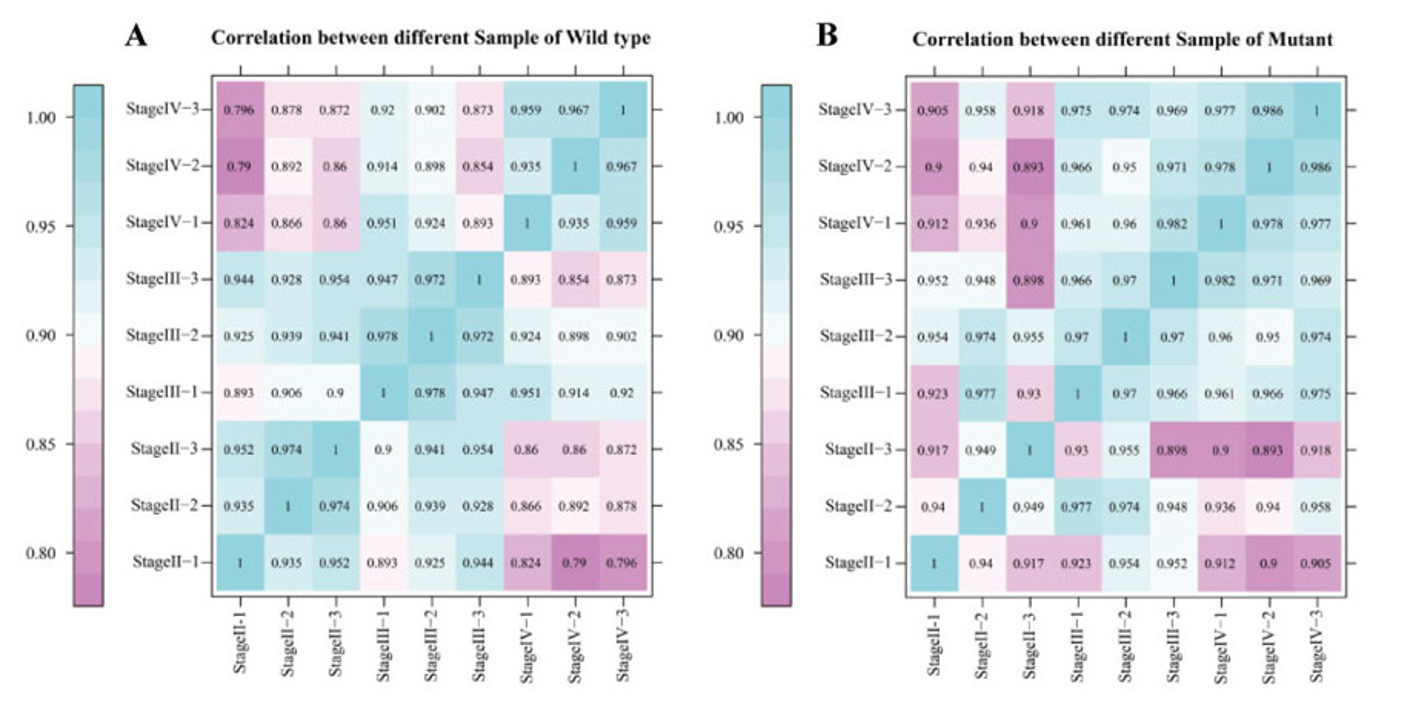

Supplement: Supplementary Table S1 — The DEGs in this study. DEG-IIs represent differentially expressed genes (DEGs) with the highest expression levels at stage II of wild type; DEG-II–IIIs represent differentially expressed genes (DEGs) that showed no significant difference in expression level between stage II and stage III but were significantly downregulated at stage IV (for convenience, we called them DEG-II–IIIs) of wild type; DEG-IVs represent differentially expressed genes (DEGs) with the highest expression levels at stage IV of wild type; u-regulation represents upregulated differentially expressed genes (DEGs) in mutant; and downregulation represents downregulated differentially expressed genes (DEGs) in mutant. [file Data_Sheet_1.ZIP › Additional files/Figure S1.png]

# Statistics of Pathway Enrichment

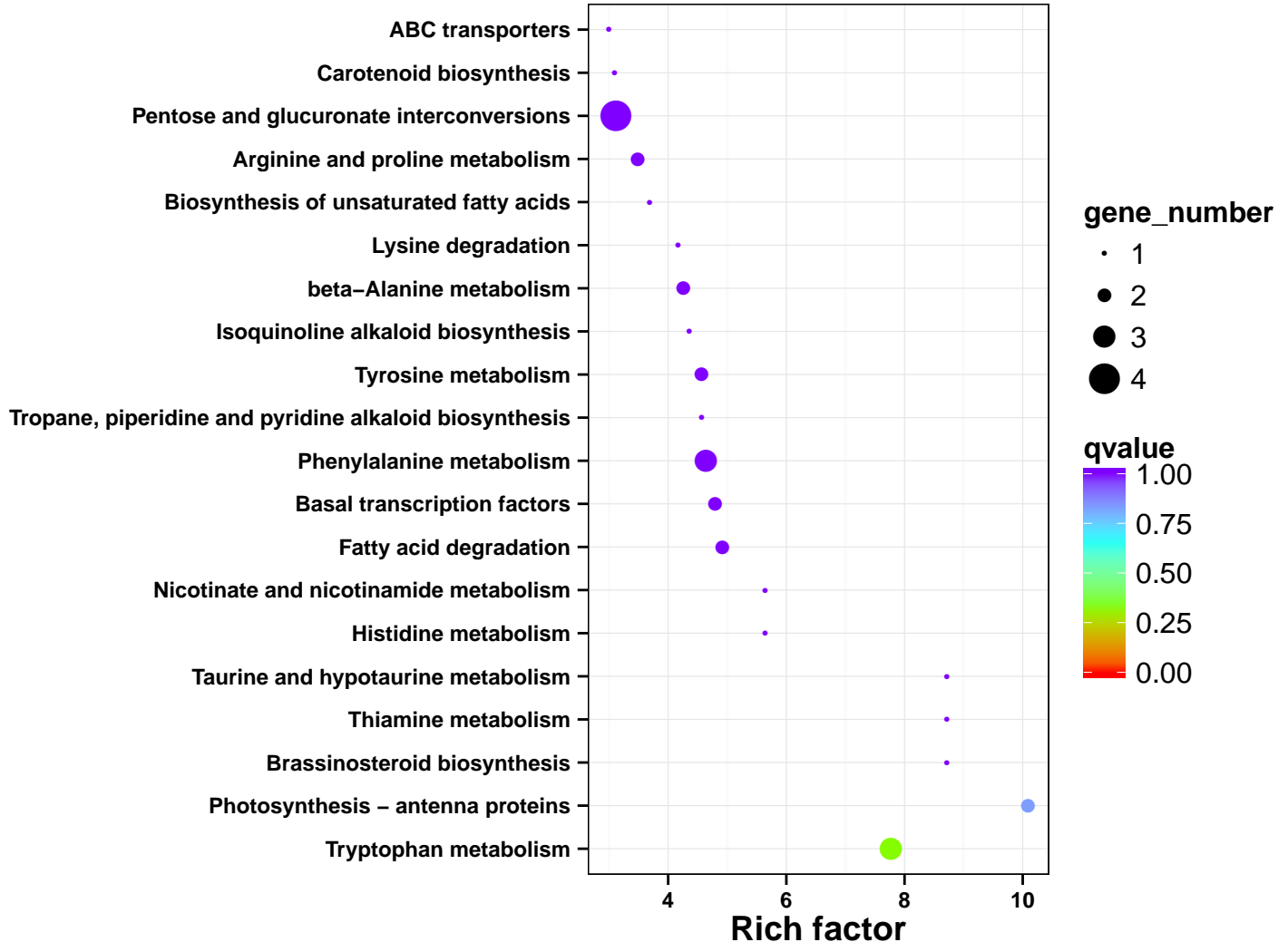

Supplement: Supplementary Table S1 — The DEGs in this study. DEG-IIs represent differentially expressed genes (DEGs) with the highest expression levels at stage II of wild type; DEG-II–IIIs represent differentially expressed genes (DEGs) that showed no significant difference in expression level between stage II and stage III but were significantly downregulated at stage IV (for convenience, we called them DEG-II–IIIs) of wild type; DEG-IVs represent differentially expressed genes (DEGs) with the highest expression levels at stage IV of wild type; u-regulation represents upregulated differentially expressed genes (DEGs) in mutant; and downregulation represents downregulated differentially expressed genes (DEGs) in mutant. [file Data_Sheet_1.ZIP › Additional files/Figure S10.pdf]

# Statistics of Pathway Enrichment

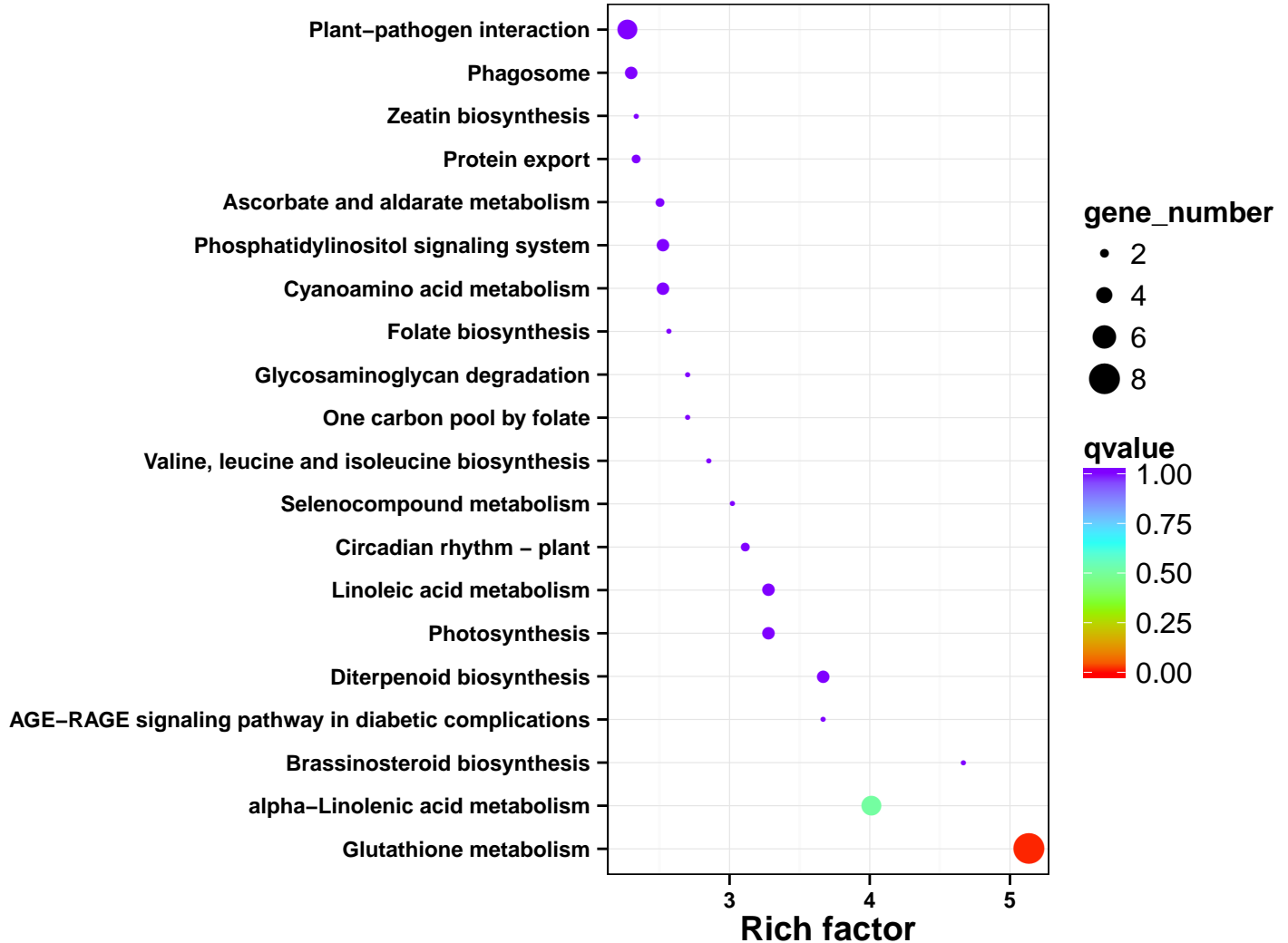

Supplement: Supplementary Table S1 — The DEGs in this study. DEG-IIs represent differentially expressed genes (DEGs) with the highest expression levels at stage II of wild type; DEG-II–IIIs represent differentially expressed genes (DEGs) that showed no significant difference in expression level between stage II and stage III but were significantly downregulated at stage IV (for convenience, we called them DEG-II–IIIs) of wild type; DEG-IVs represent differentially expressed genes (DEGs) with the highest expression levels at stage IV of wild type; u-regulation represents upregulated differentially expressed genes (DEGs) in mutant; and downregulation represents downregulated differentially expressed genes (DEGs) in mutant. [file Data_Sheet_1.ZIP › Additional files/Figure S11.pdf]

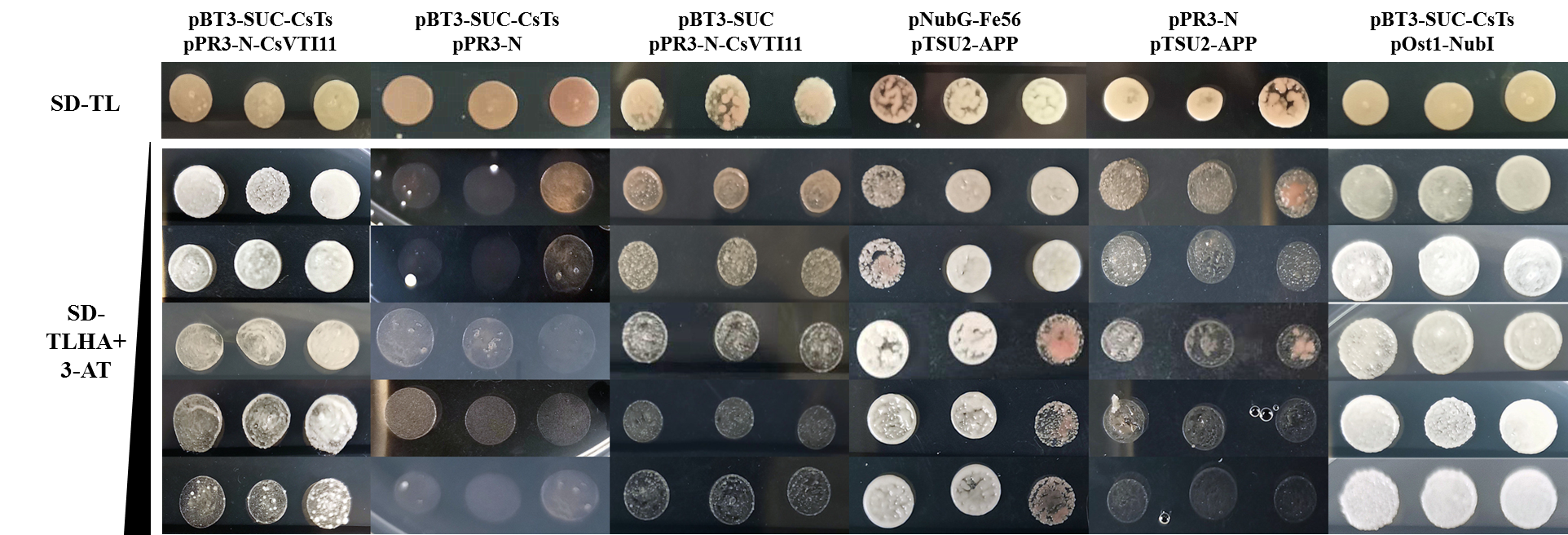

Supplement: Supplementary Table S1 — The DEGs in this study. DEG-IIs represent differentially expressed genes (DEGs) with the highest expression levels at stage II of wild type; DEG-II–IIIs represent differentially expressed genes (DEGs) that showed no significant difference in expression level between stage II and stage III but were significantly downregulated at stage IV (for convenience, we called them DEG-II–IIIs) of wild type; DEG-IVs represent differentially expressed genes (DEGs) with the highest expression levels at stage IV of wild type; u-regulation represents upregulated differentially expressed genes (DEGs) in mutant; and downregulation represents downregulated differentially expressed genes (DEGs) in mutant. [file Data_Sheet_1.ZIP › Additional files/Figure S12.png]

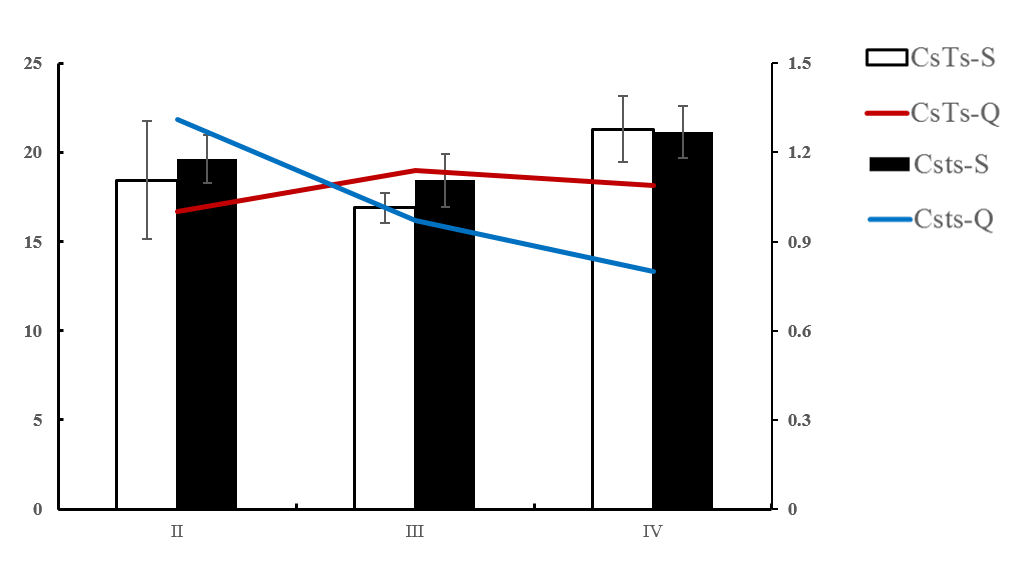

Supplement: Supplementary Table S1 — The DEGs in this study. DEG-IIs represent differentially expressed genes (DEGs) with the highest expression levels at stage II of wild type; DEG-II–IIIs represent differentially expressed genes (DEGs) that showed no significant difference in expression level between stage II and stage III but were significantly downregulated at stage IV (for convenience, we called them DEG-II–IIIs) of wild type; DEG-IVs represent differentially expressed genes (DEGs) with the highest expression levels at stage IV of wild type; u-regulation represents upregulated differentially expressed genes (DEGs) in mutant; and downregulation represents downregulated differentially expressed genes (DEGs) in mutant. [file Data_Sheet_1.ZIP › Additional files/Figure S2.png]

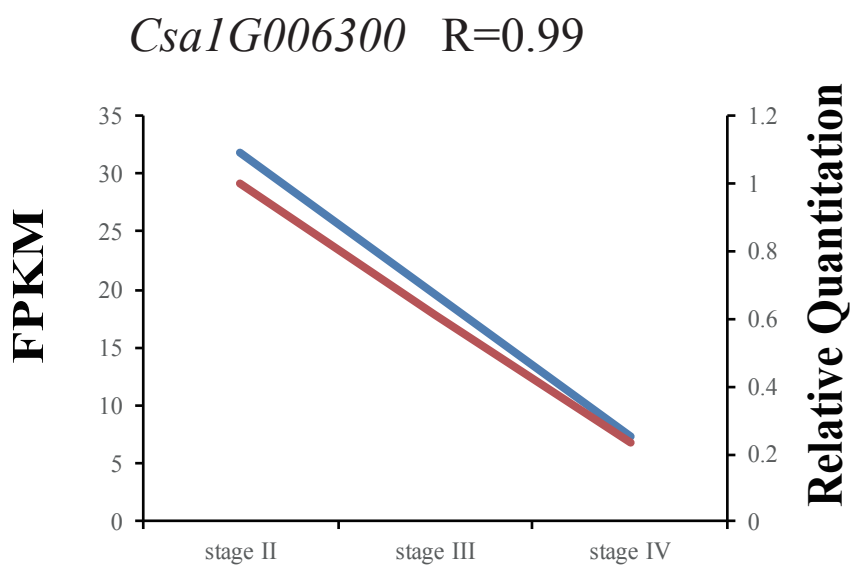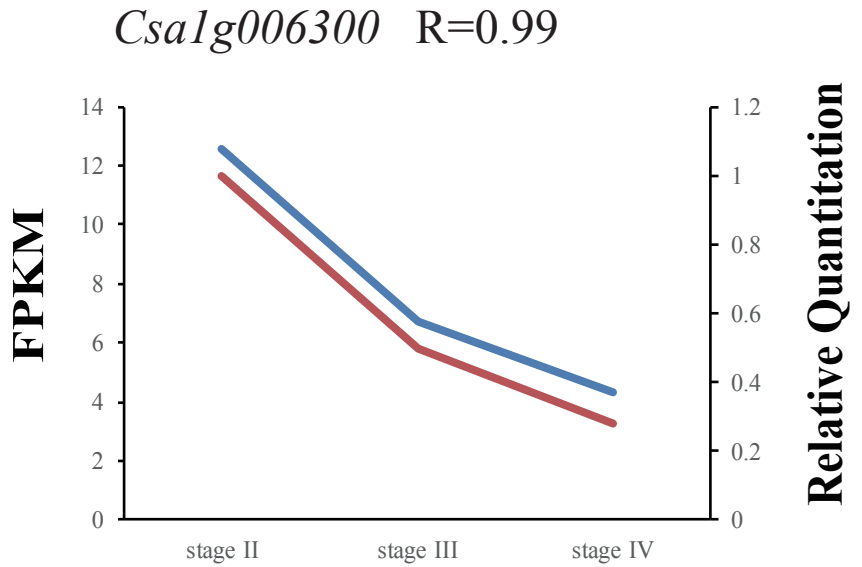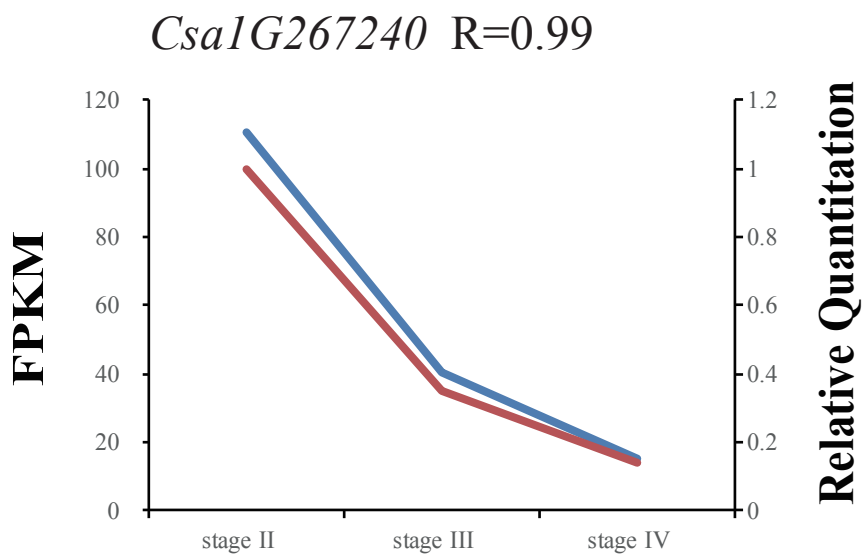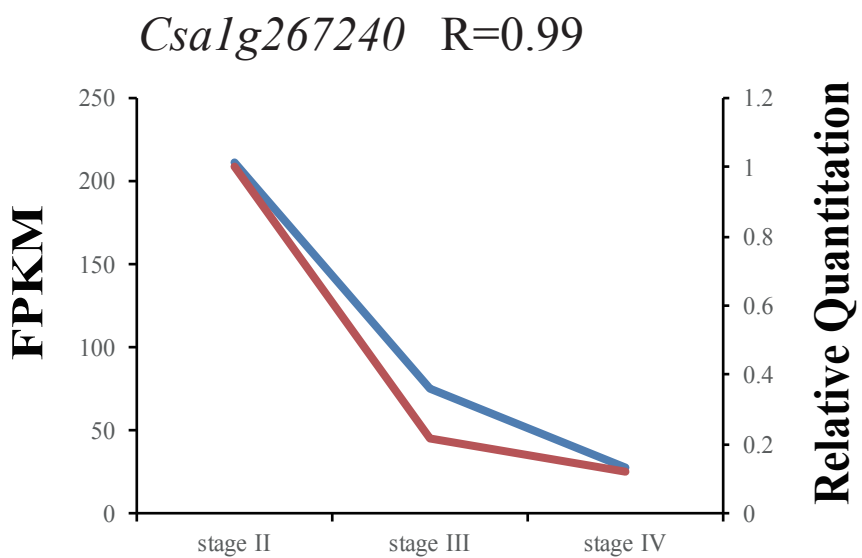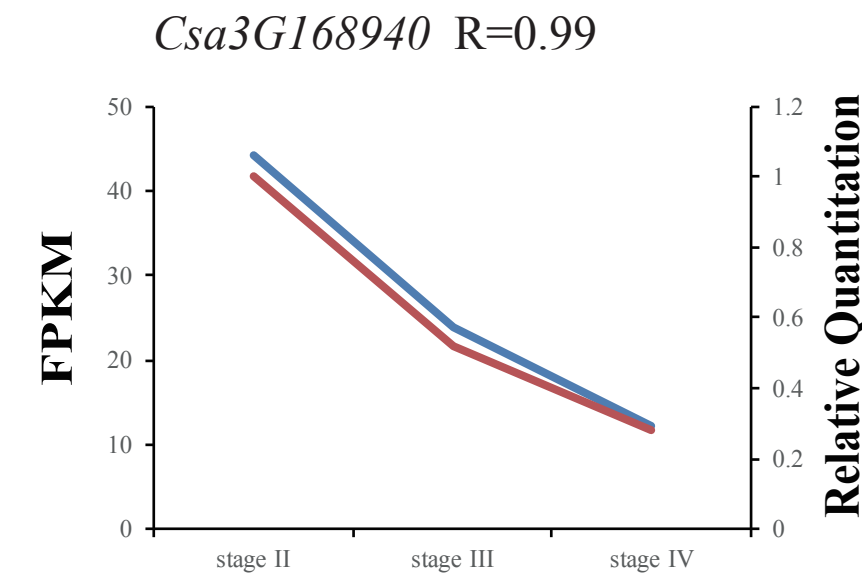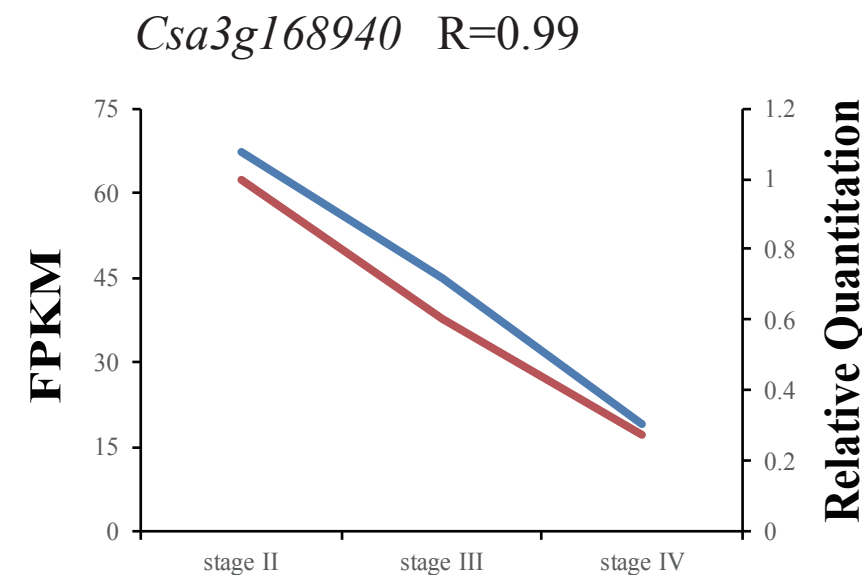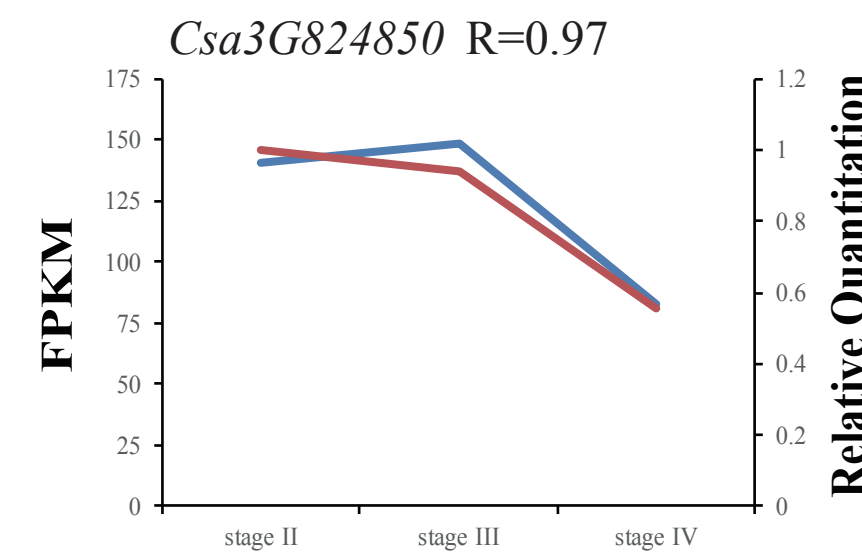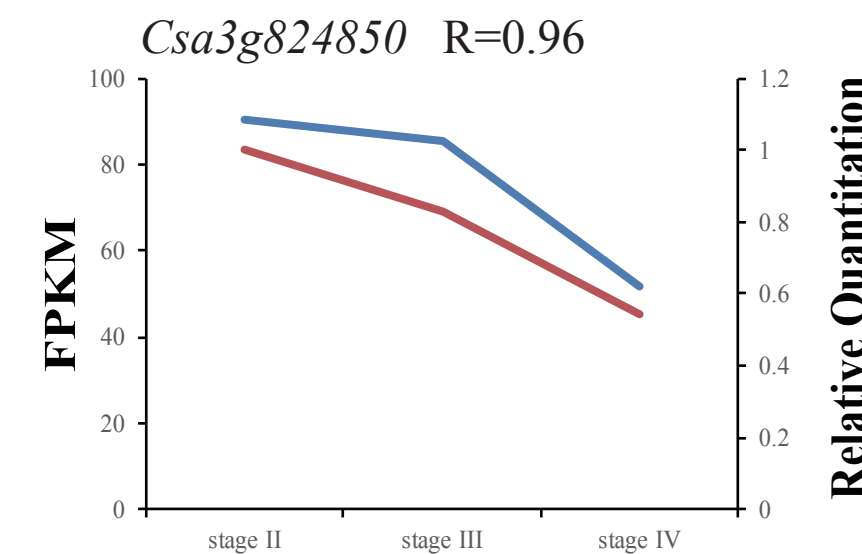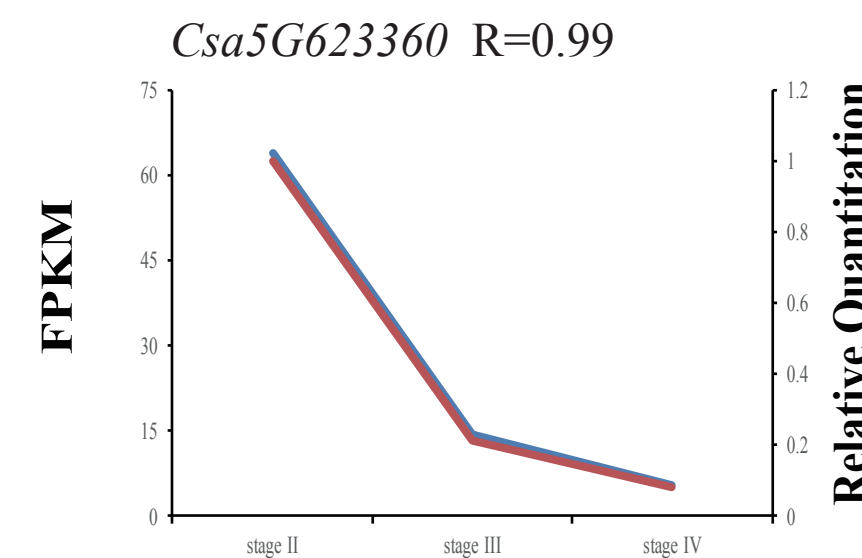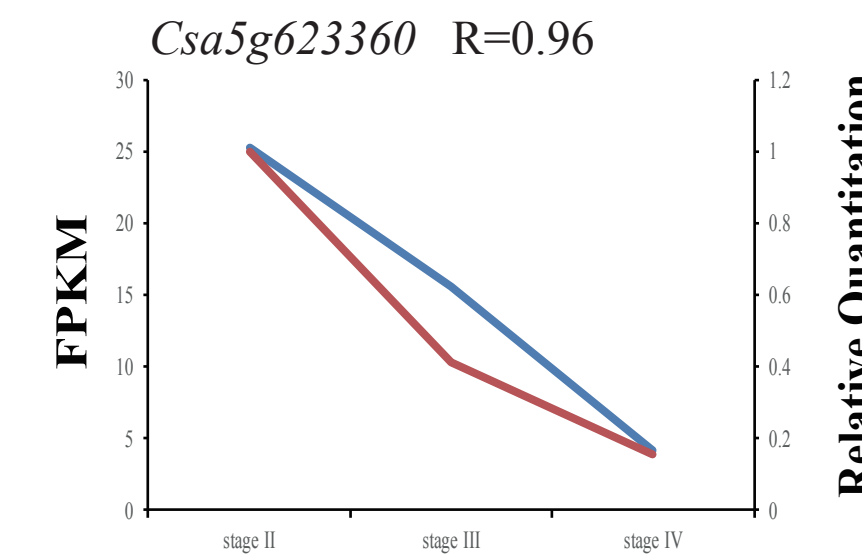

*CsTu* R=0.99

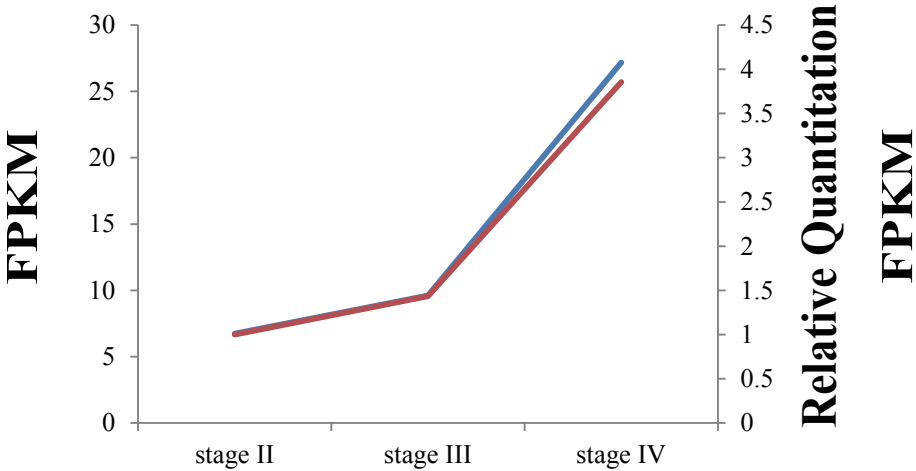

*Cstu* R=0.99

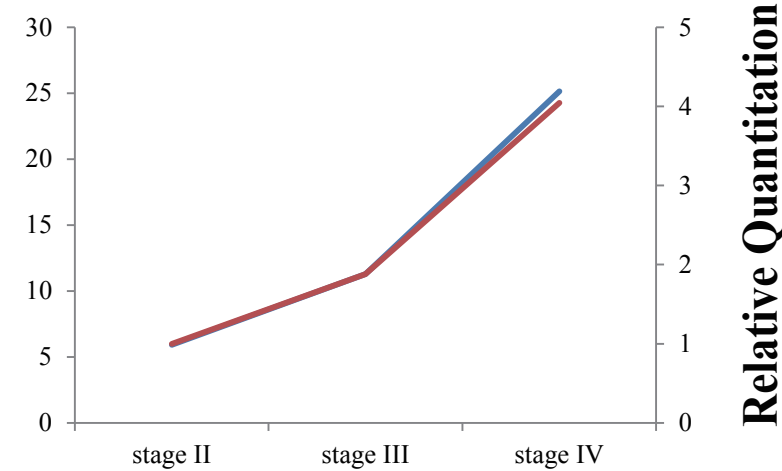

*CsMYB6* R=0.99

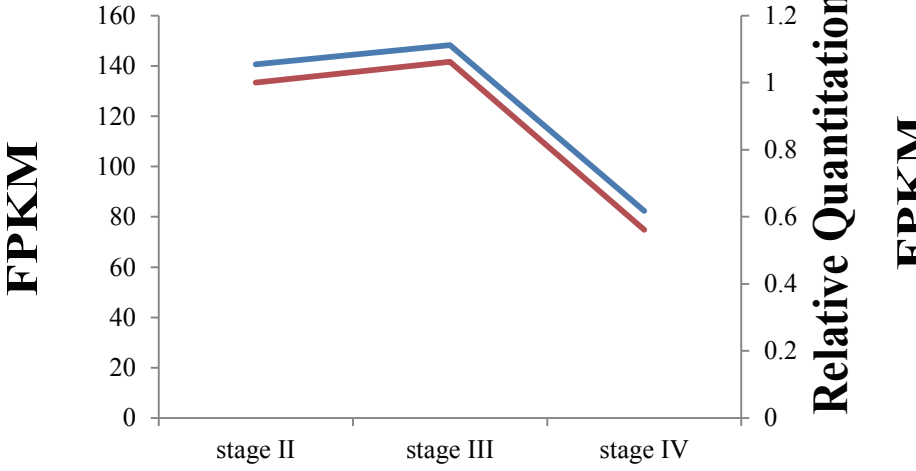

*Csmyb6* R=0.99

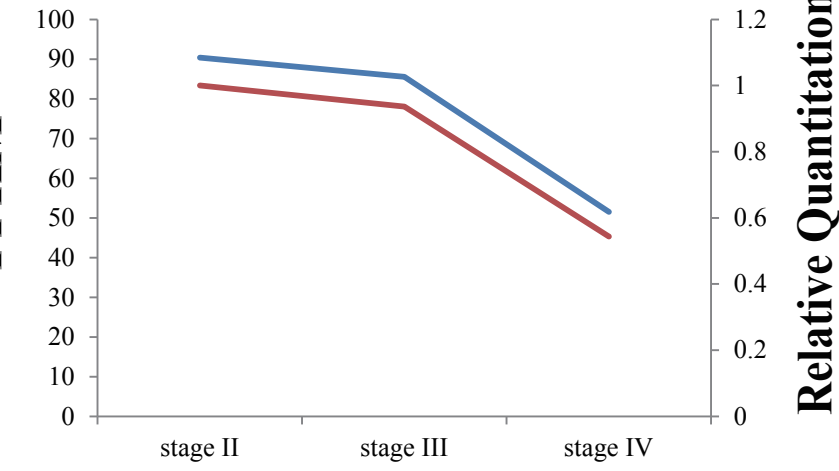

*CsMict* R=0.99

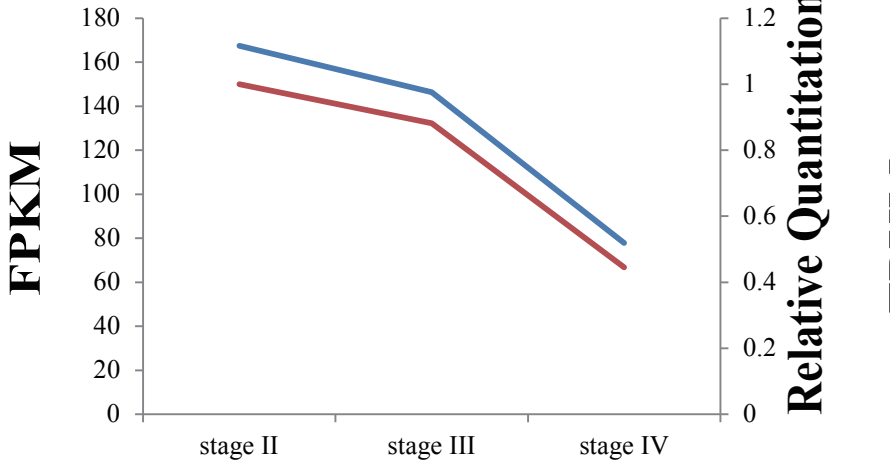

*Csmict* R=0.99

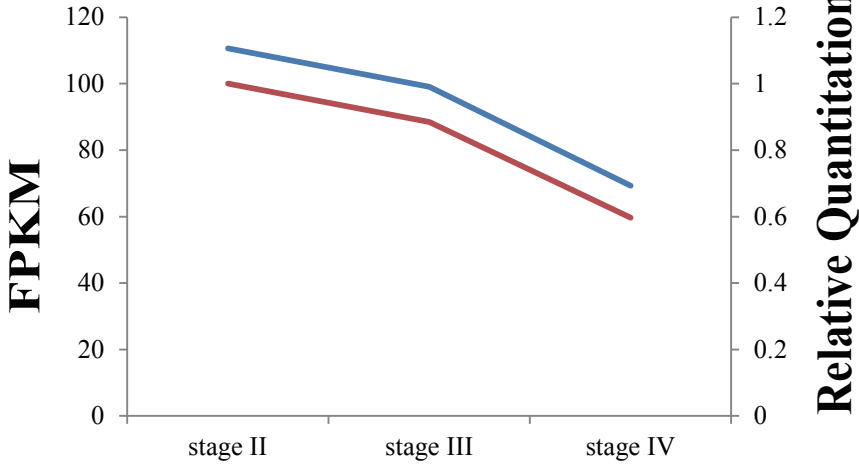

*Csa3G020060* R=0.99

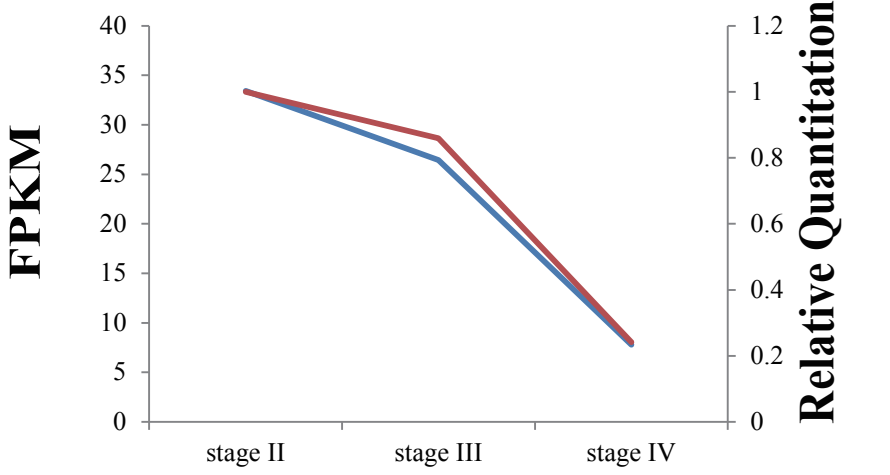

*Csa3g020060* R=0.99

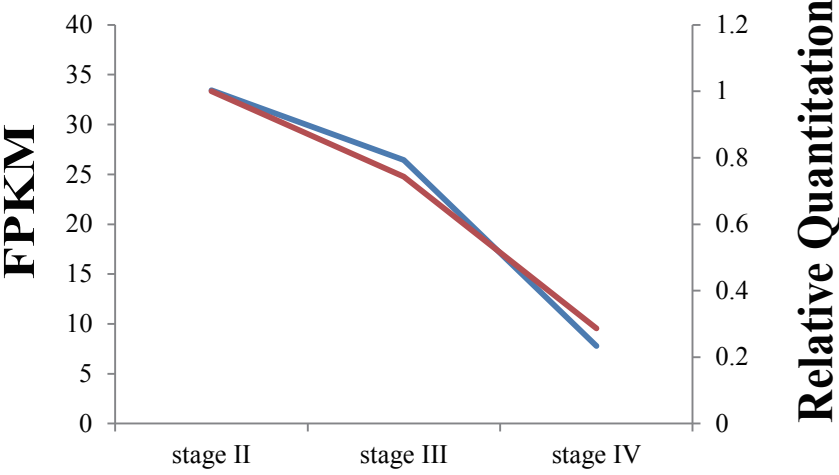

*Csa2G006270* R=0.98

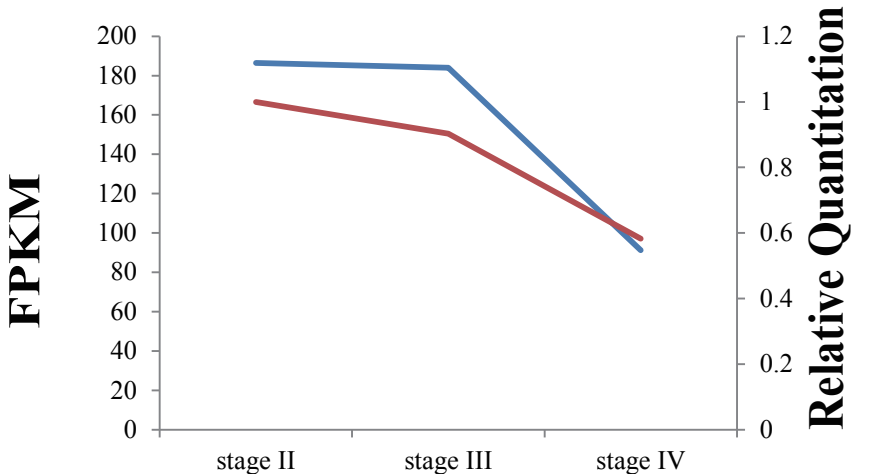

*Csa2g006270* R=0.82

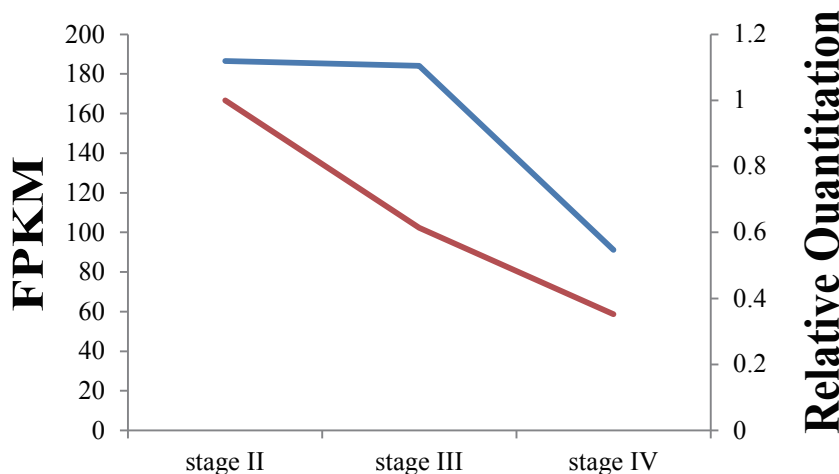

Supplement: Supplementary Table S1 — The DEGs in this study. DEG-IIs represent differentially expressed genes (DEGs) with the highest expression levels at stage II of wild type; DEG-II–IIIs represent differentially expressed genes (DEGs) that showed no significant difference in expression level between stage II and stage III but were significantly downregulated at stage IV (for convenience, we called them DEG-II–IIIs) of wild type; DEG-IVs represent differentially expressed genes (DEGs) with the highest expression levels at stage IV of wild type; u-regulation represents upregulated differentially expressed genes (DEGs) in mutant; and downregulation represents downregulated differentially expressed genes (DEGs) in mutant. [file Data_Sheet_1.ZIP › Additional files/Figure S3.pdf]

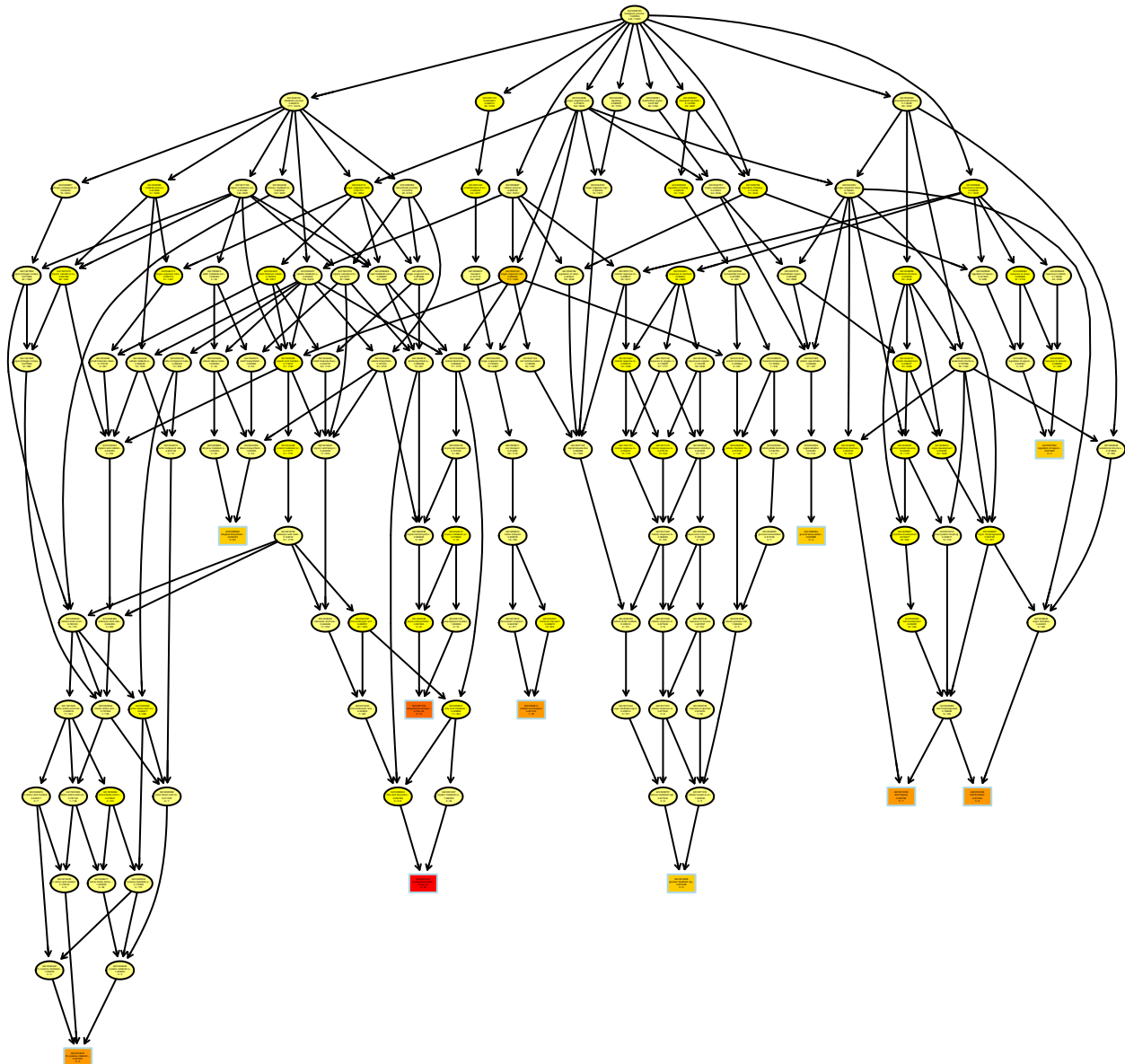

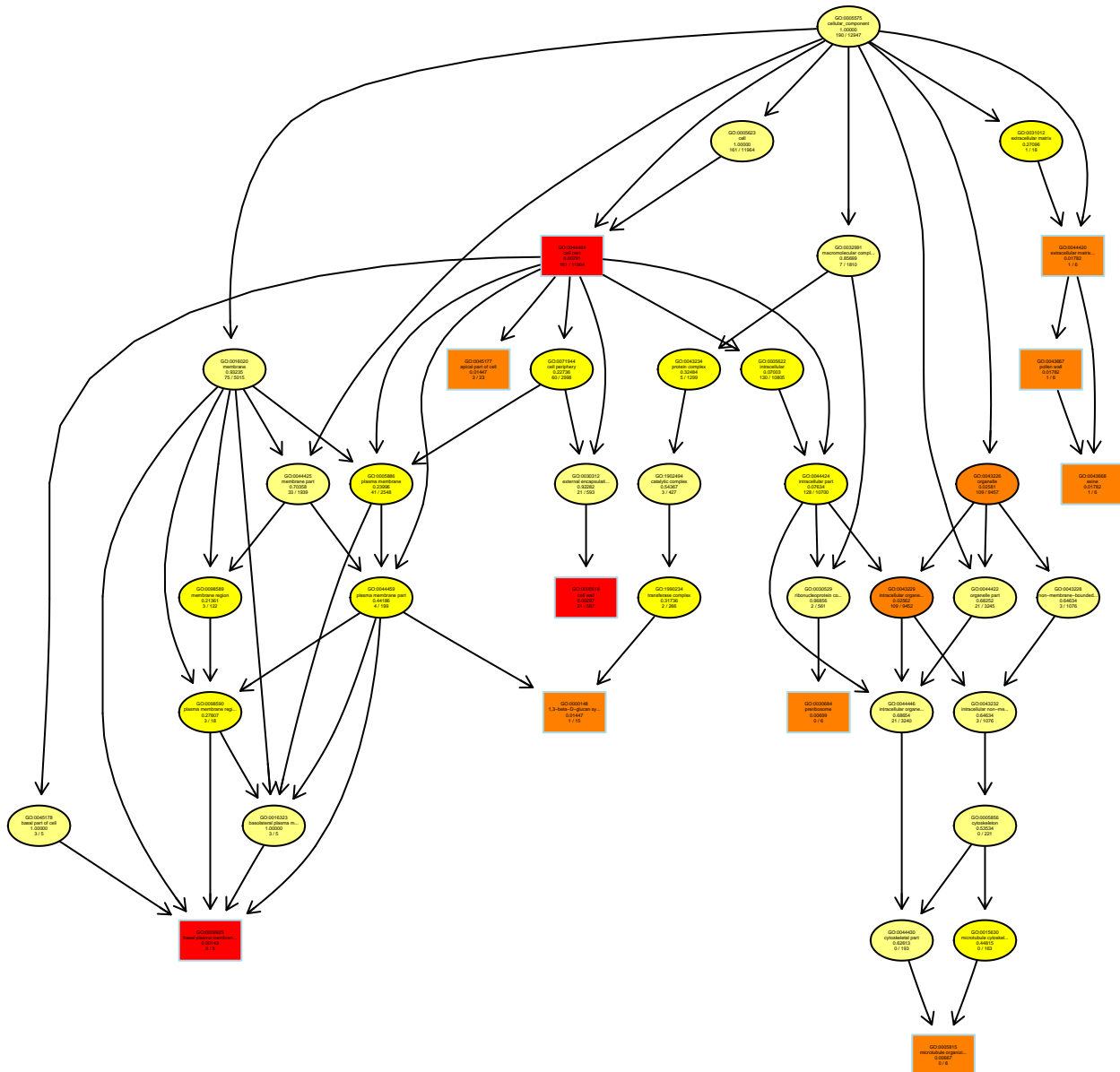

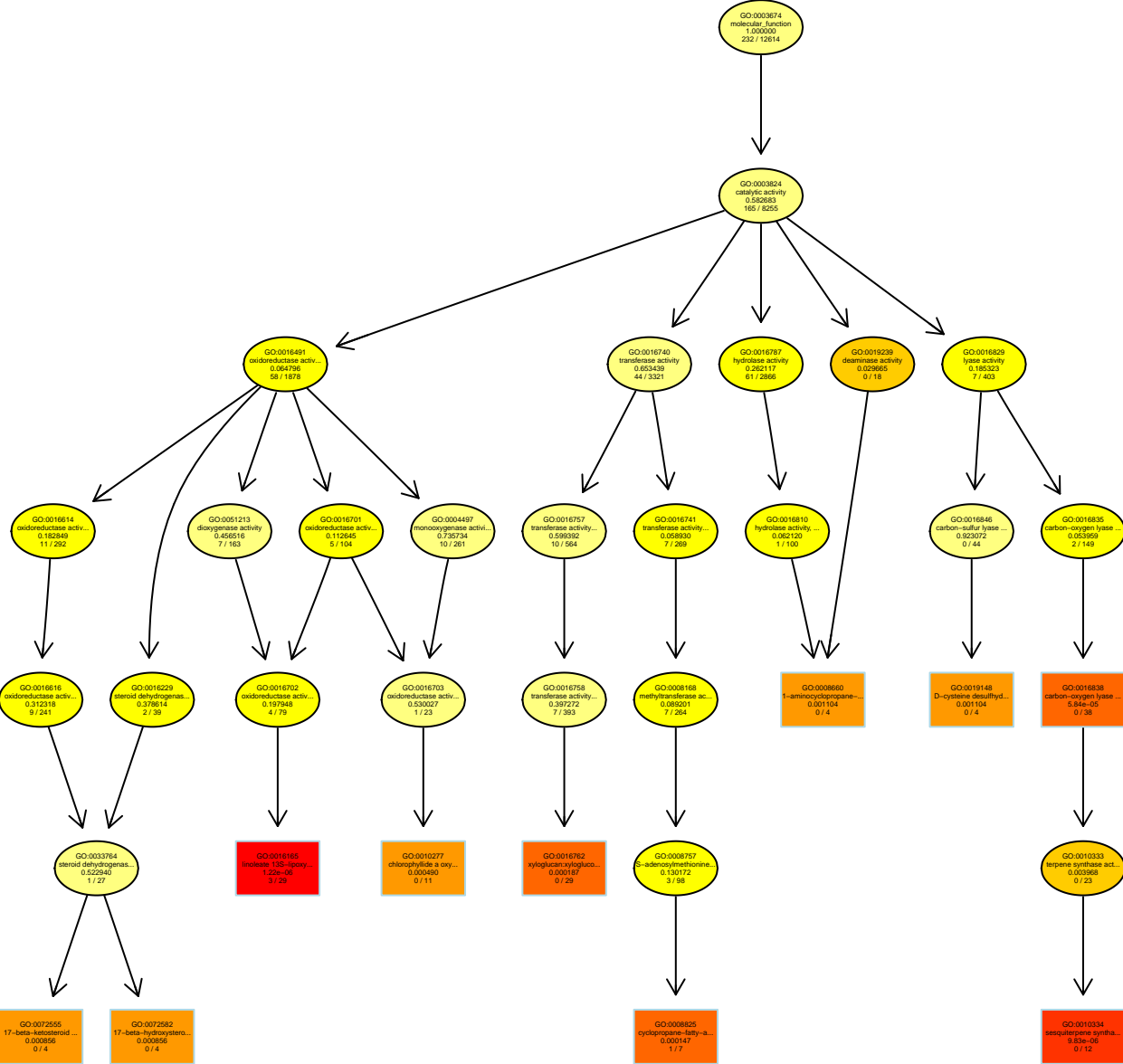

Supplement: Supplementary Table S1 — The DEGs in this study. DEG-IIs represent differentially expressed genes (DEGs) with the highest expression levels at stage II of wild type; DEG-II–IIIs represent differentially expressed genes (DEGs) that showed no significant difference in expression level between stage II and stage III but were significantly downregulated at stage IV (for convenience, we called them DEG-II–IIIs) of wild type; DEG-IVs represent differentially expressed genes (DEGs) with the highest expression levels at stage IV of wild type; u-regulation represents upregulated differentially expressed genes (DEGs) in mutant; and downregulation represents downregulated differentially expressed genes (DEGs) in mutant. [file Data_Sheet_1.ZIP › Additional files/Figure S4.pdf]

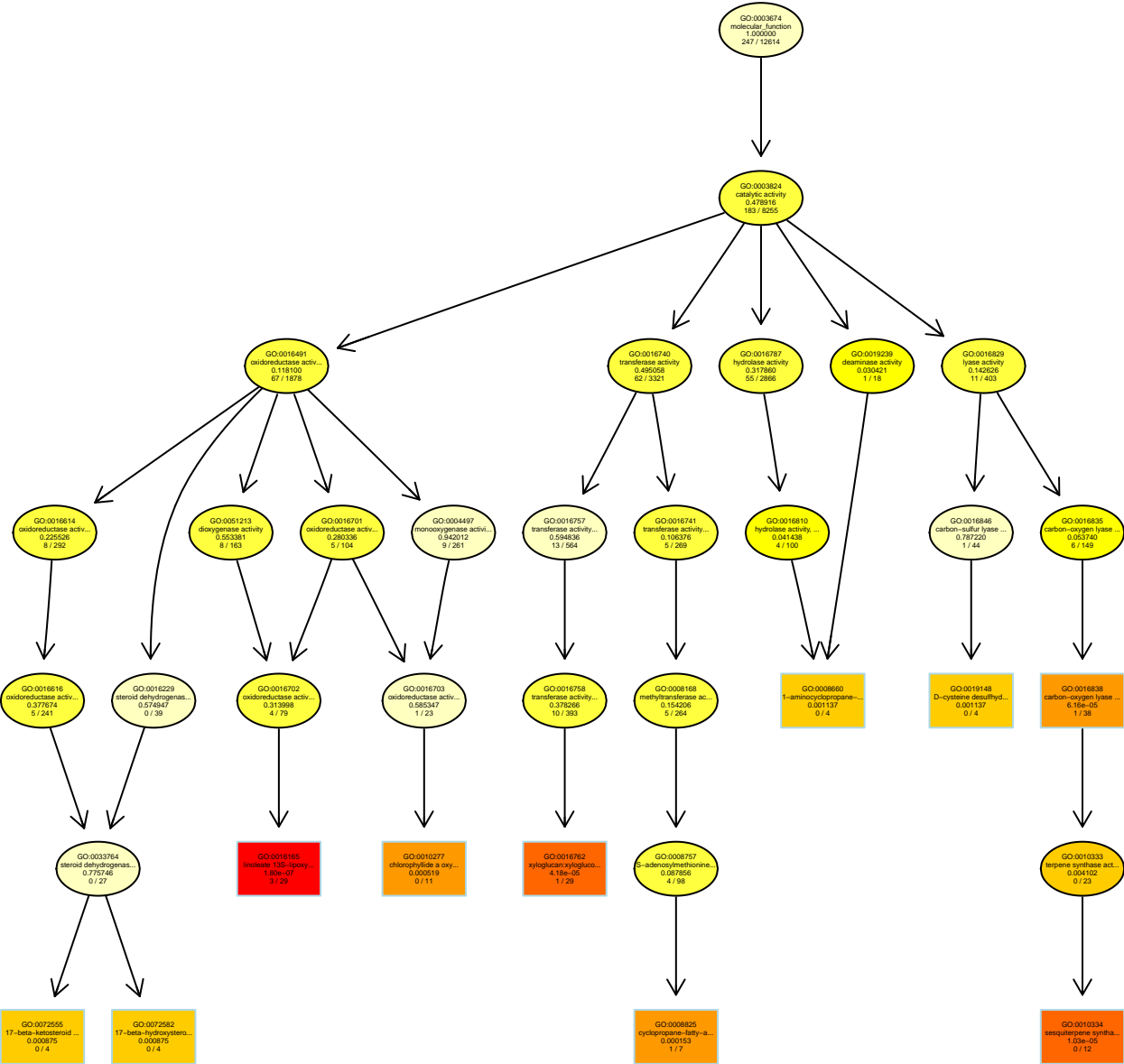

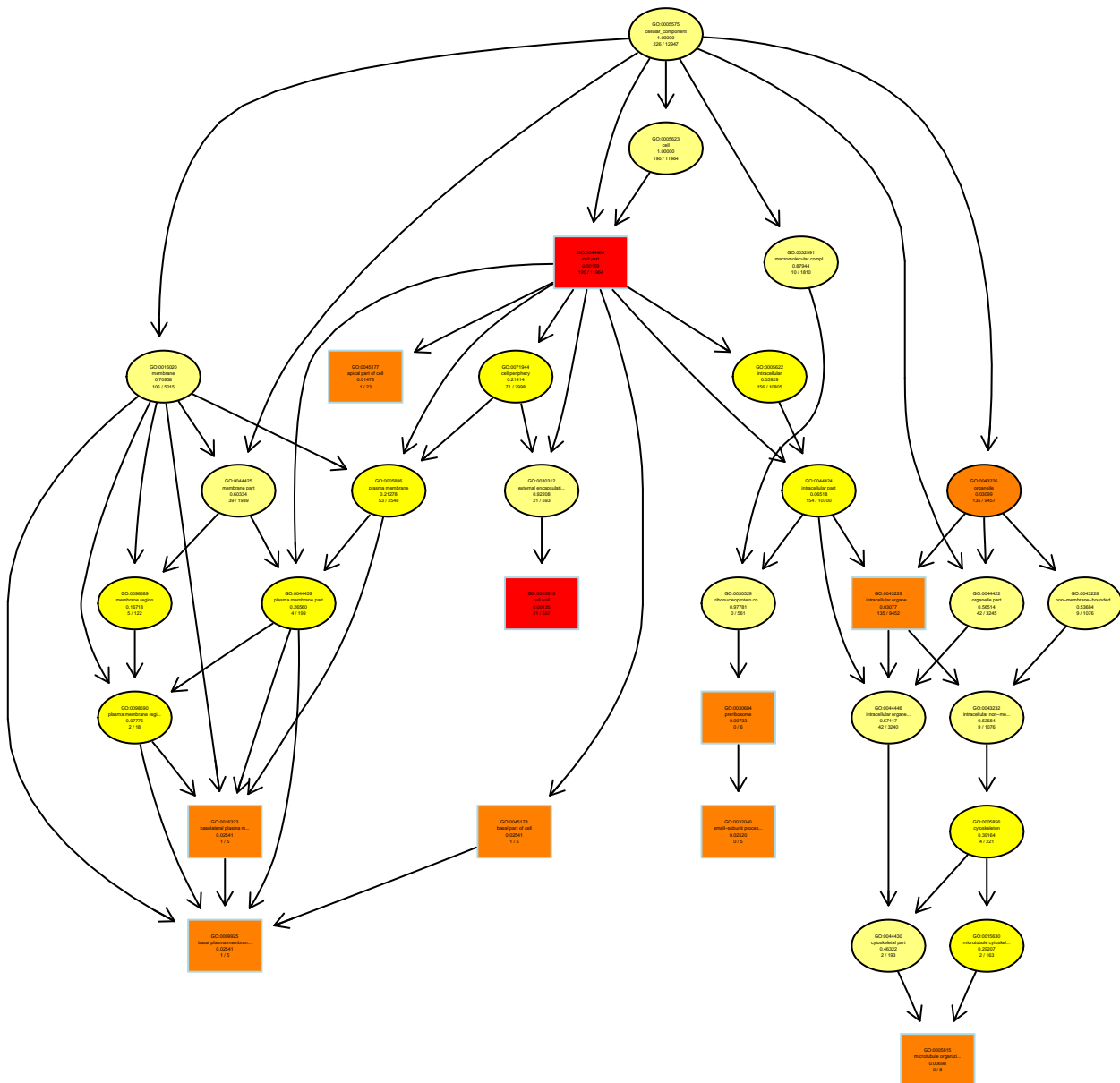

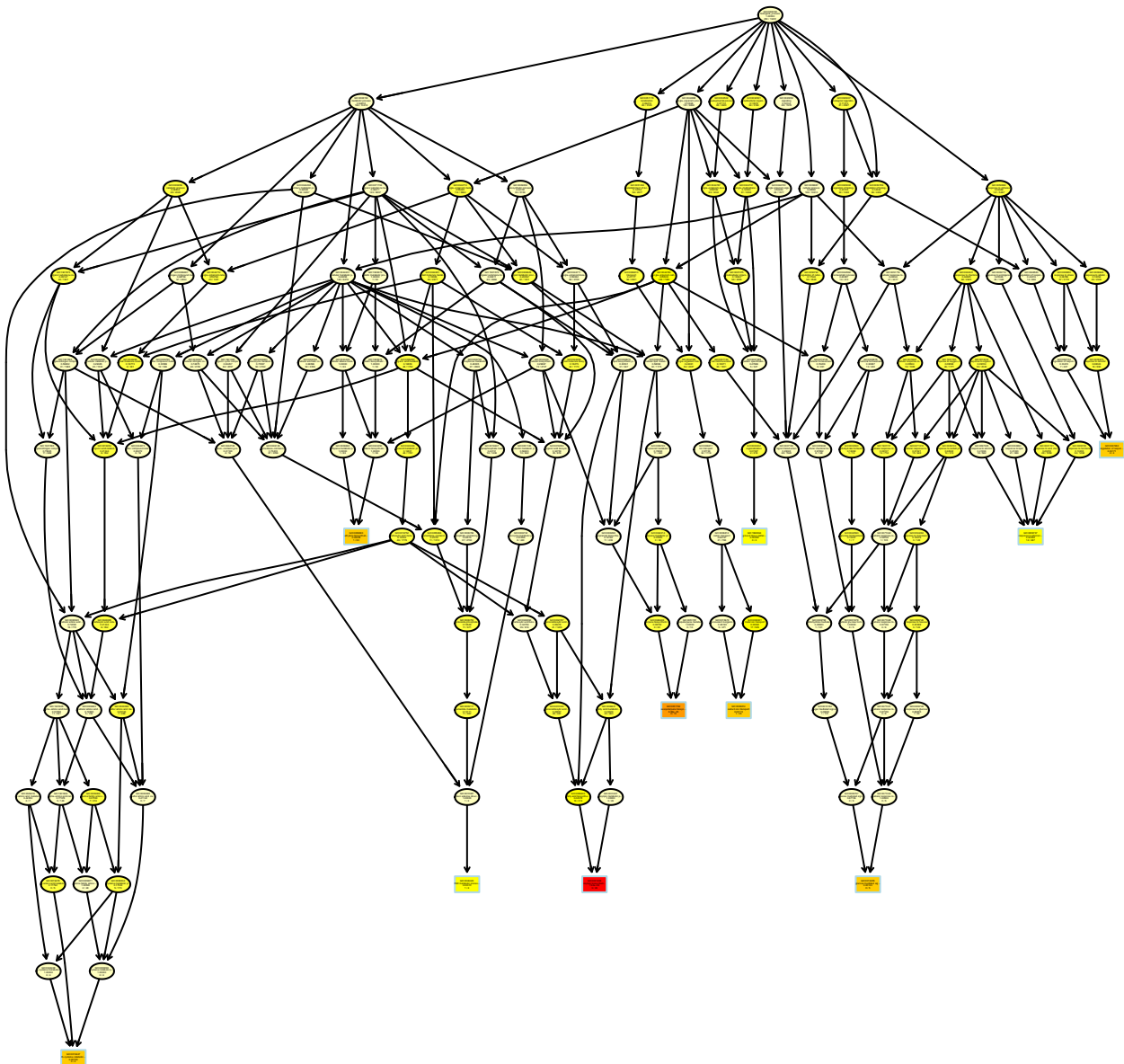

Supplement: Supplementary Table S1 — The DEGs in this study. DEG-IIs represent differentially expressed genes (DEGs) with the highest expression levels at stage II of wild type; DEG-II–IIIs represent differentially expressed genes (DEGs) that showed no significant difference in expression level between stage II and stage III but were significantly downregulated at stage IV (for convenience, we called them DEG-II–IIIs) of wild type; DEG-IVs represent differentially expressed genes (DEGs) with the highest expression levels at stage IV of wild type; u-regulation represents upregulated differentially expressed genes (DEGs) in mutant; and downregulation represents downregulated differentially expressed genes (DEGs) in mutant. [file Data_Sheet_1.ZIP › Additional files/Figure S6.pdf]

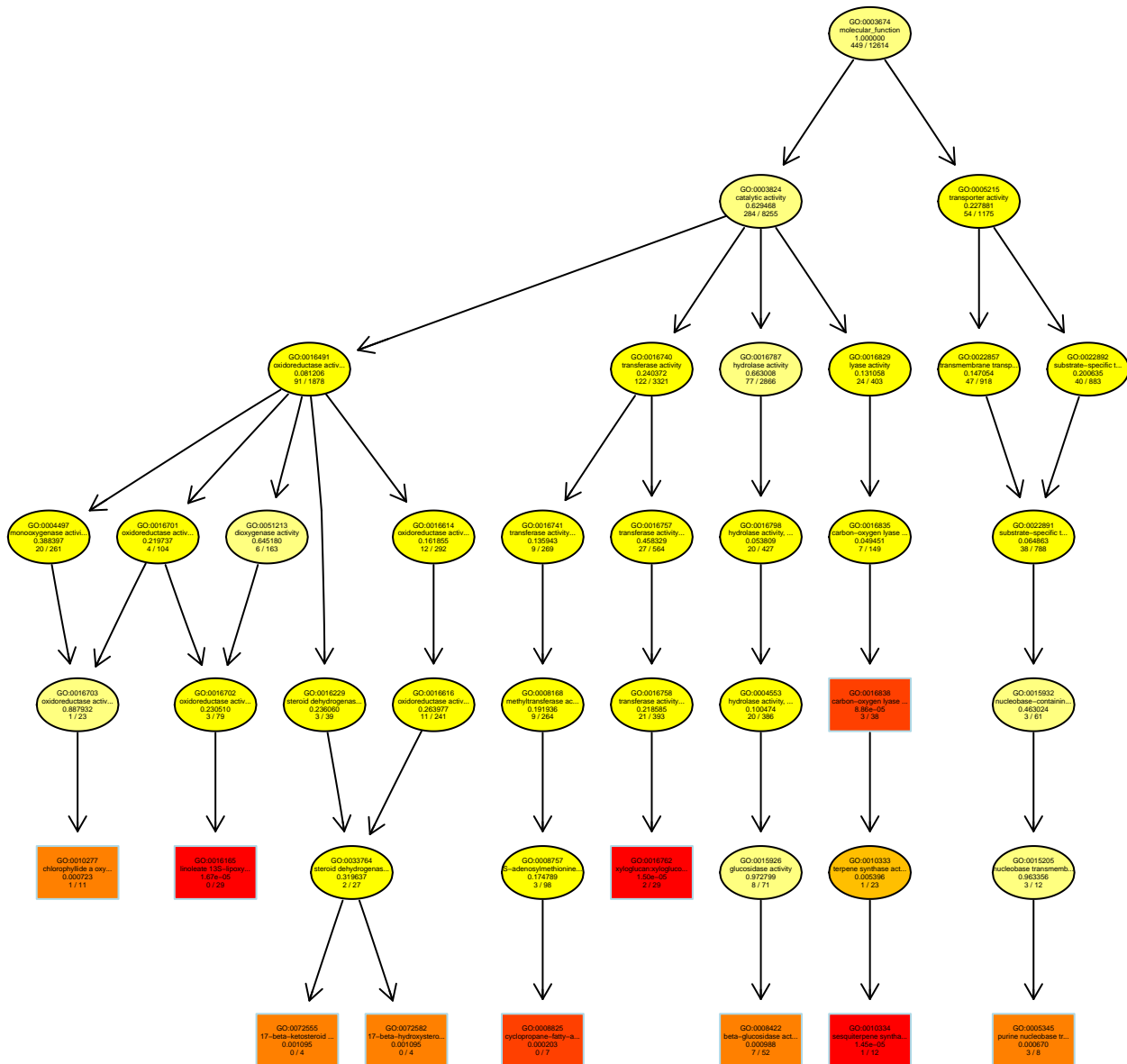

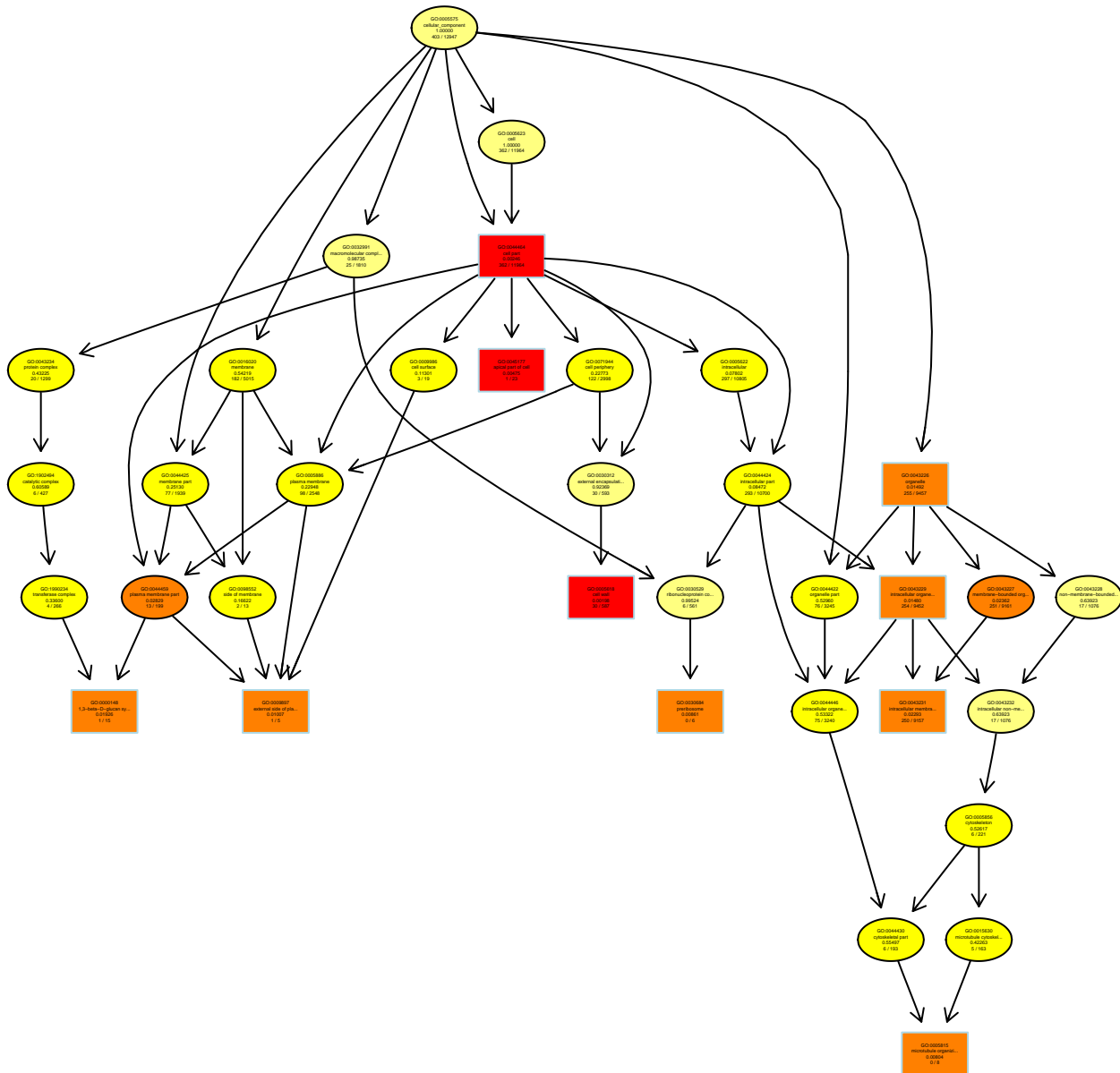

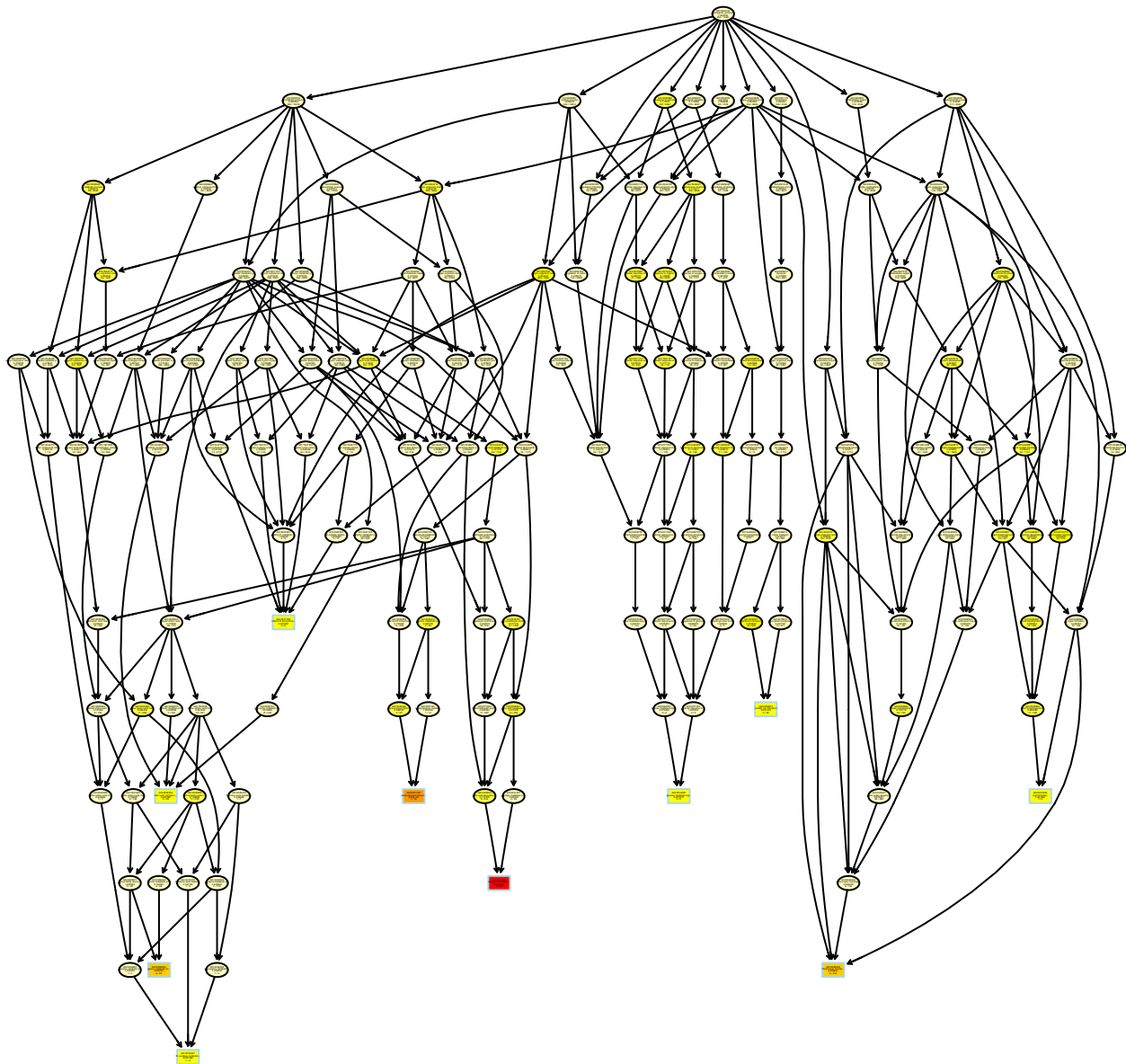

Supplement: Supplementary Table S1 — The DEGs in this study. DEG-IIs represent differentially expressed genes (DEGs) with the highest expression levels at stage II of wild type; DEG-II–IIIs represent differentially expressed genes (DEGs) that showed no significant difference in expression level between stage II and stage III but were significantly downregulated at stage IV (for convenience, we called them DEG-II–IIIs) of wild type; DEG-IVs represent differentially expressed genes (DEGs) with the highest expression levels at stage IV of wild type; u-regulation represents upregulated differentially expressed genes (DEGs) in mutant; and downregulation represents downregulated differentially expressed genes (DEGs) in mutant. [file Data_Sheet_1.ZIP › Additional files/Figure S7.pdf]

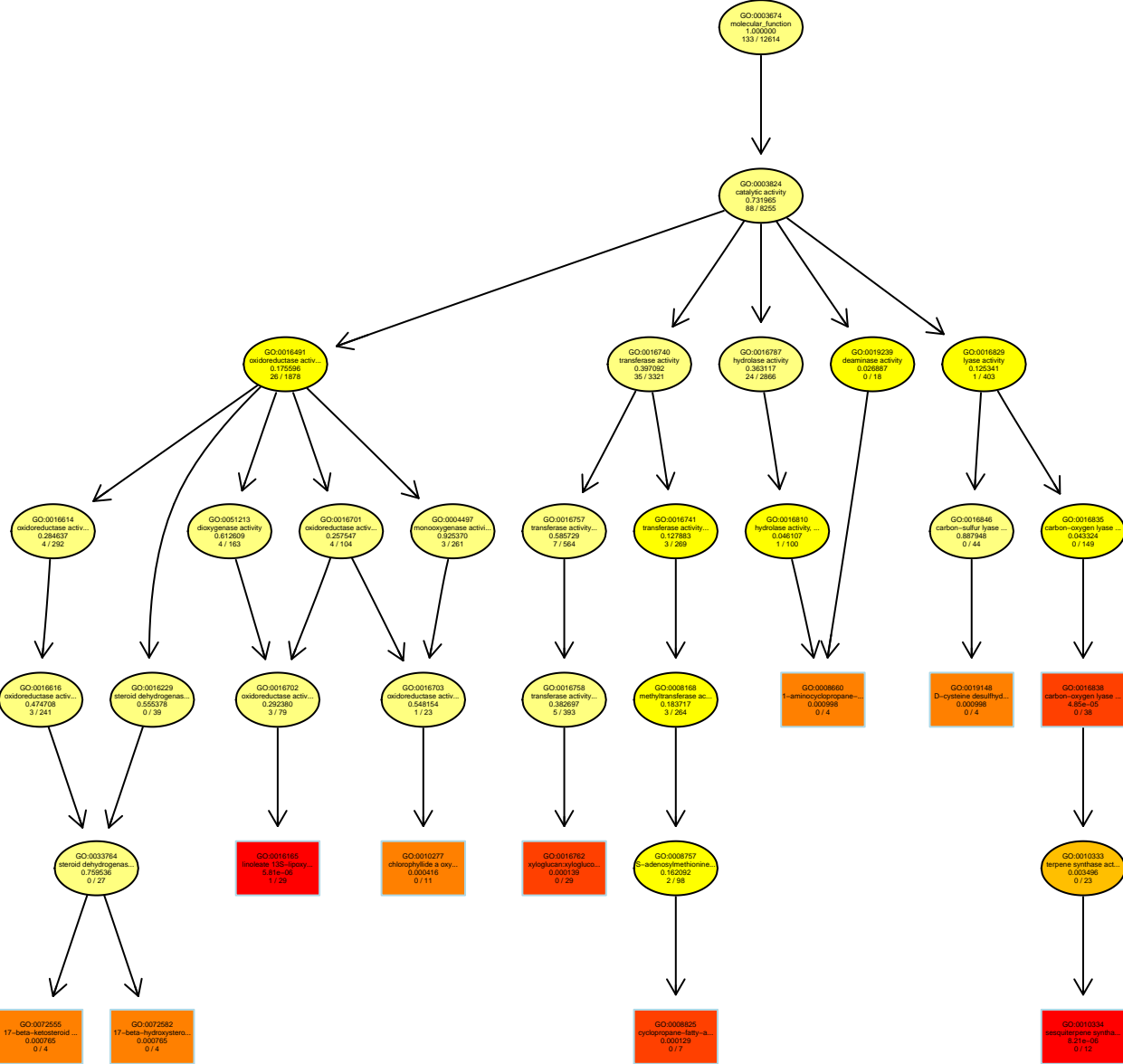

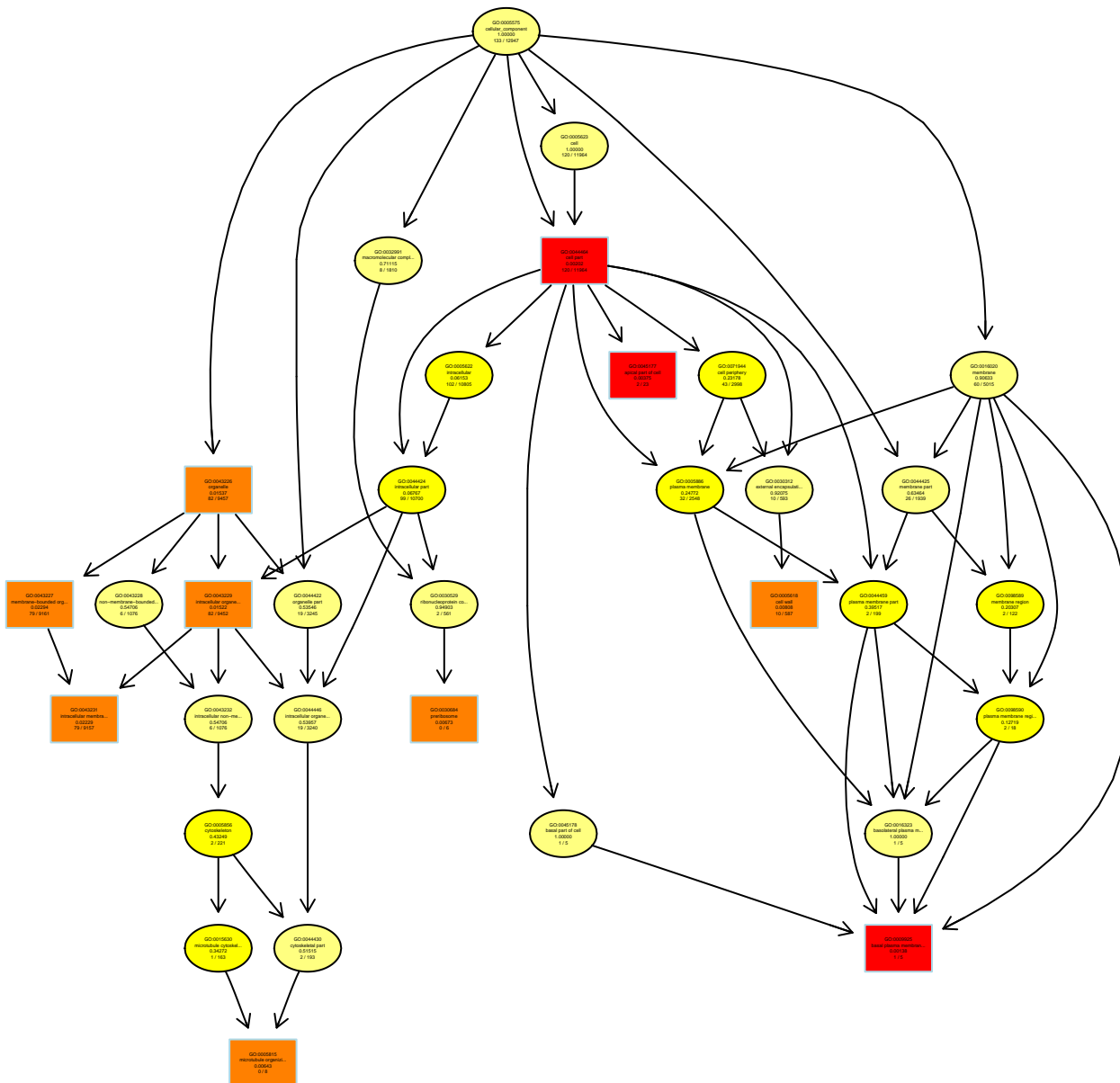

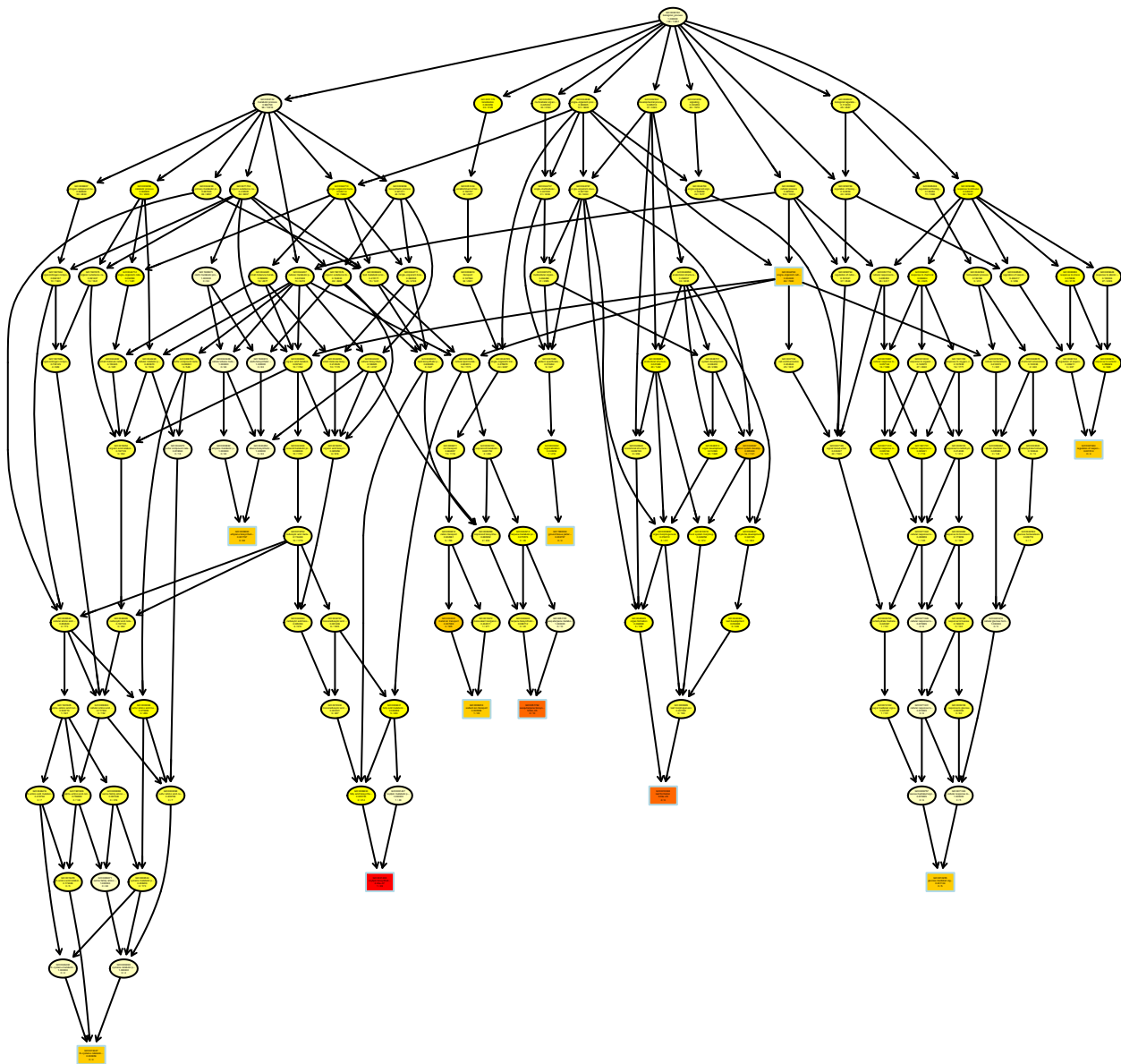

Supplement: Supplementary Table S1 — The DEGs in this study. DEG-IIs represent differentially expressed genes (DEGs) with the highest expression levels at stage II of wild type; DEG-II–IIIs represent differentially expressed genes (DEGs) that showed no significant difference in expression level between stage II and stage III but were significantly downregulated at stage IV (for convenience, we called them DEG-II–IIIs) of wild type; DEG-IVs represent differentially expressed genes (DEGs) with the highest expression levels at stage IV of wild type; u-regulation represents upregulated differentially expressed genes (DEGs) in mutant; and downregulation represents downregulated differentially expressed genes (DEGs) in mutant. [file Data_Sheet_1.ZIP › Additional files/Figure S8.pdf]

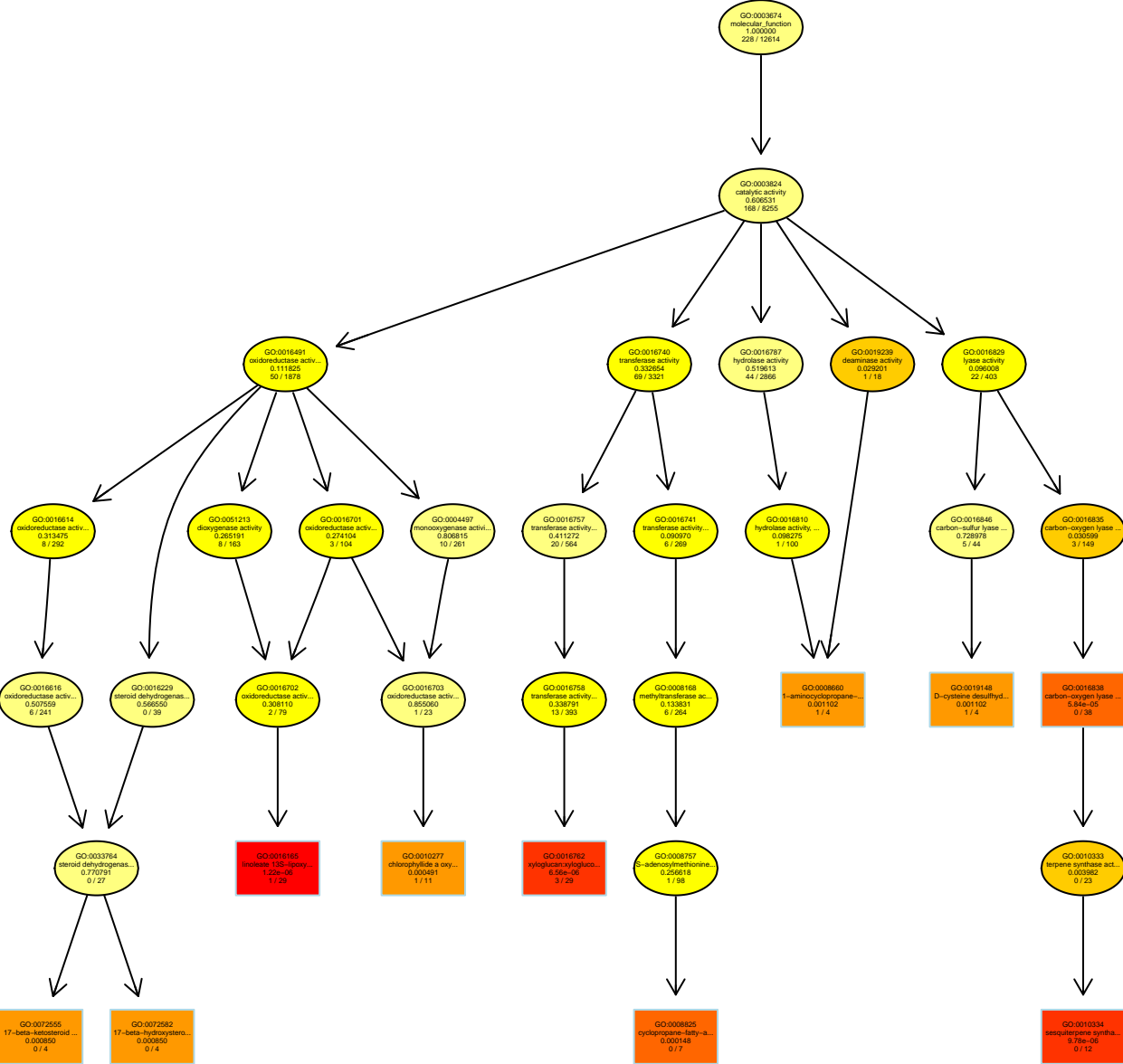

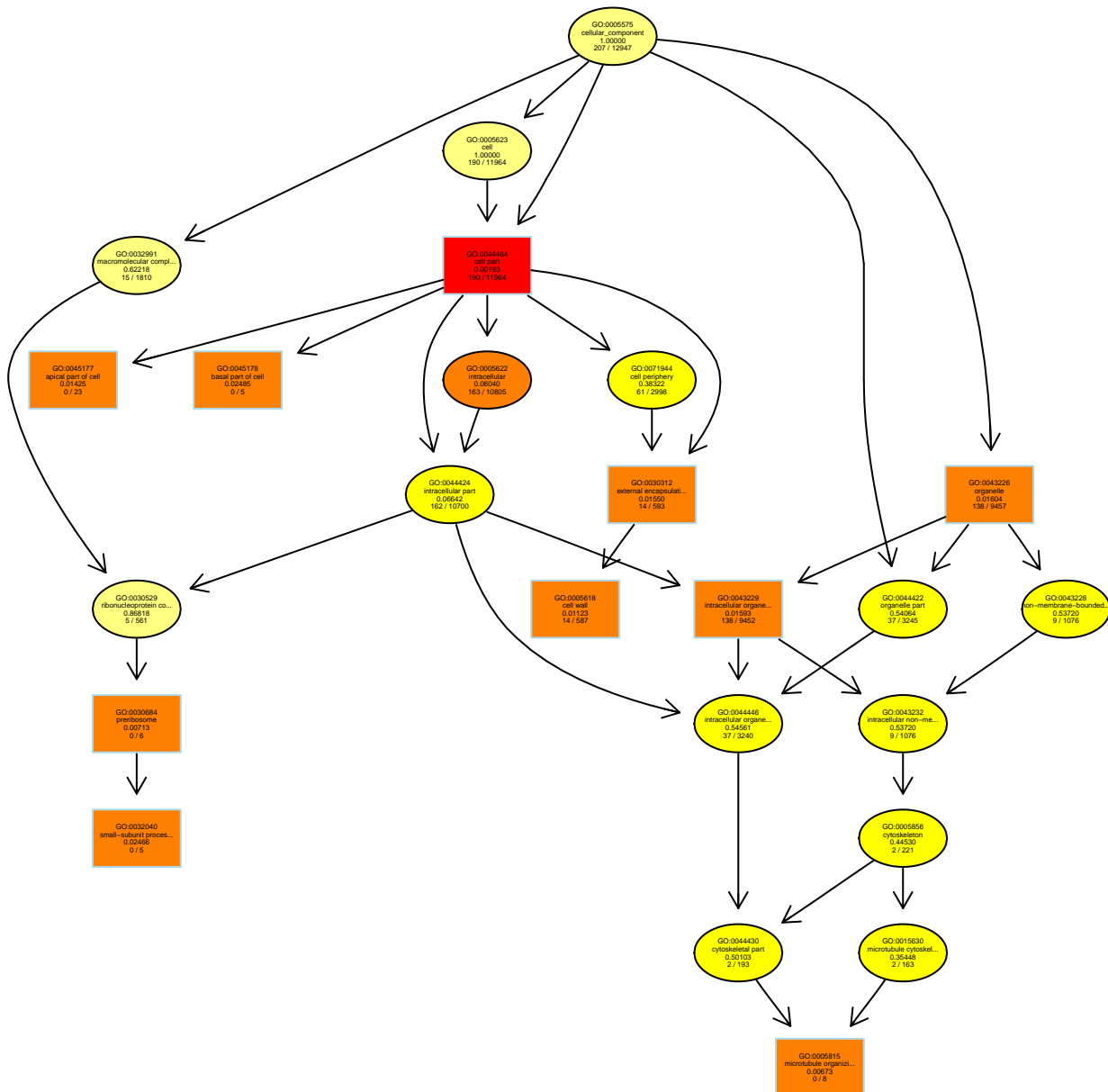

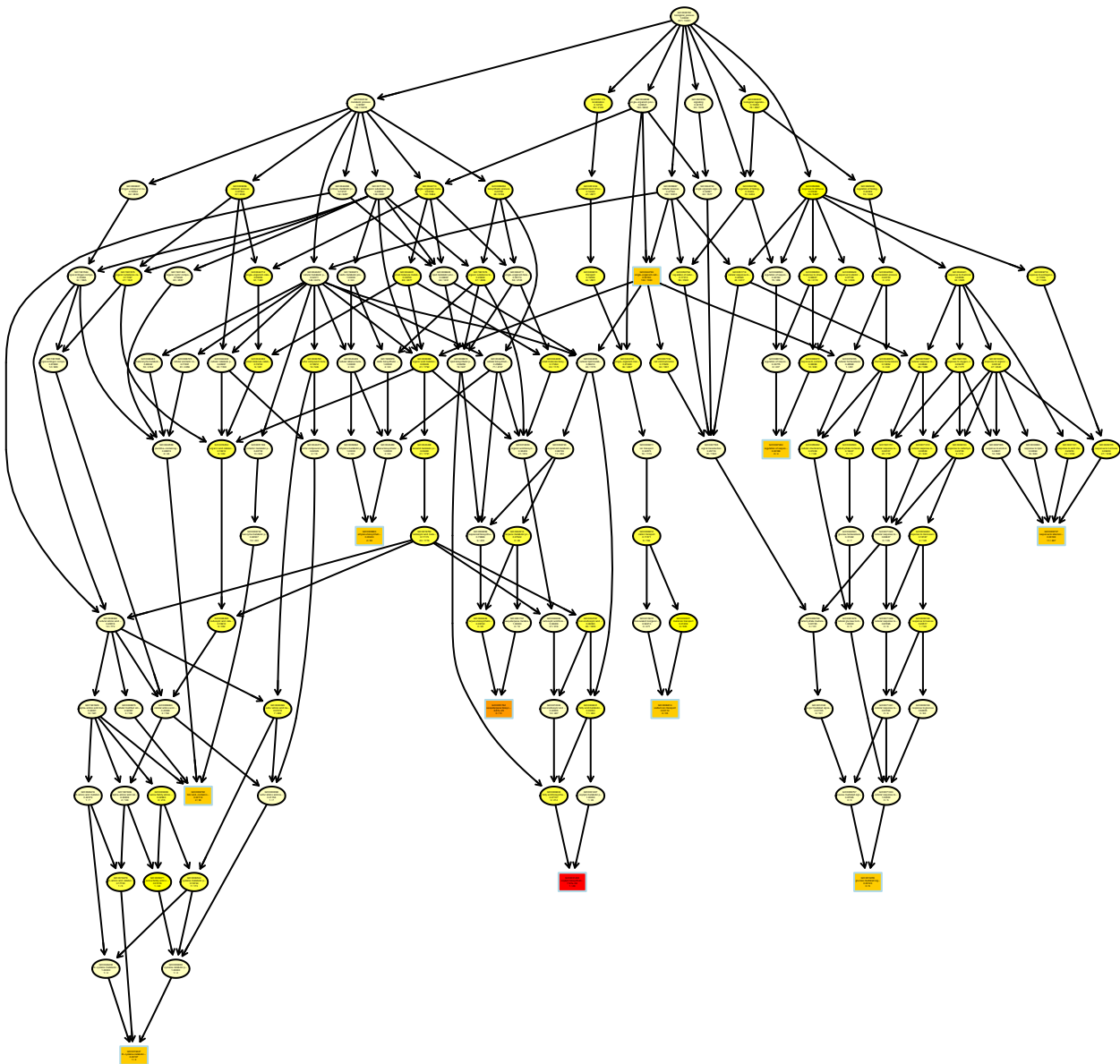

Supplement: Supplementary Table S1 — The DEGs in this study. DEG-IIs represent differentially expressed genes (DEGs) with the highest expression levels at stage II of wild type; DEG-II–IIIs represent differentially expressed genes (DEGs) that showed no significant difference in expression level between stage II and stage III but were significantly downregulated at stage IV (for convenience, we called them DEG-II–IIIs) of wild type; DEG-IVs represent differentially expressed genes (DEGs) with the highest expression levels at stage IV of wild type; u-regulation represents upregulated differentially expressed genes (DEGs) in mutant; and downregulation represents downregulated differentially expressed genes (DEGs) in mutant. [file Data_Sheet_1.ZIP › Additional files/Figure S9.pdf]
